# Targeting Mre11 overcomes platinum resistance and induces synthetic lethality in XRCC1 deficient epithelial ovarian cancers

**DOI:** 10.1038/s41698-022-00298-0

**Published:** 2022-07-19

**Authors:** Adel Alblihy, Reem Ali, Mashael Algethami, Ahmed Shoqafi, Michael S. Toss, Juliette Brownlie, Natalie J. Tatum, Ian Hickson, Paloma Ordonez Moran, Anna Grabowska, Jennie N. Jeyapalan, Nigel P. Mongan, Emad A. Rakha, Srinivasan Madhusudan

**Affiliations:** 1 Nottingham Biodiscovery Institute, School of Medicine, University of Nottingham, University Park, Nottingham, NG7 3RD UK; 2 Medical Center, King Fahad Security College (KFSC), Riyadh, 11461 Saudi Arabia; 3 Department of Pathology, Nottingham University Hospitals, City Hospital Campus, Nottingham, NG5 1PB UK; 4 Cancer Research UK Newcastle Drug Discovery Unit, Translational and Clinical Research Institute, Newcastle University Centre for Cancer, Faculty of Medical Sciences, Newcastle University, Newcastle upon Tyne, UK; 5 Department of Pharmacology, Weill Cornell Medicine, New York, 10065 NY USA; 6 Department of Oncology, Nottingham University Hospitals, Nottingham, NG51PB UK

**Keywords:** Ovarian cancer, Ovarian cancer

## Abstract

Platinum resistance is a clinical challenge in ovarian cancer. Platinating agents induce DNA damage which activate Mre11 nuclease directed DNA damage signalling and response (DDR). Upregulation of DDR may promote chemotherapy resistance. Here we have comprehensively evaluated Mre11 in epithelial ovarian cancers. In clinical cohort that received platinum- based chemotherapy (*n* = 331), Mre11 protein overexpression was associated with aggressive phenotype and poor progression free survival (PFS) (*p* = 0.002). In the ovarian cancer genome atlas (TCGA) cohort (*n* = 498), *Mre11* gene amplification was observed in a subset of serous tumours (5%) which correlated highly with *Mre11* mRNA levels (*p* < 0.0001). Altered *Mre11* levels was linked with genome wide alterations that can influence platinum sensitivity. At the transcriptomic level (*n* = 1259), *Mre11* overexpression was associated with poor PFS (*p* = 0.003). ROC analysis showed an area under the curve (AUC) of 0.642 for response to platinum-based chemotherapy. Pre-clinically, Mre11 depletion by gene knock down or blockade by small molecule inhibitor (Mirin) reversed platinum resistance in ovarian cancer cells and in 3D spheroid models. Importantly, Mre11 inhibition was synthetically lethal in platinum sensitive XRCC1 deficient ovarian cancer cells and 3D-spheroids. Selective cytotoxicity was associated with DNA double strand break (DSB) accumulation, S-phase cell cycle arrest and increased apoptosis. We conclude that pharmaceutical development of Mre11 inhibitors is a viable clinical strategy for platinum sensitization and synthetic lethality in ovarian cancer.

## Introduction

Platinum based chemotherapy is central to ovarian cancer therapy. However, the development of intrinsic or acquired resistance adversely impacts overall survival in patients^1–3^. Platinum-induced intra-strand crosslinks adducts (which comprises about 90% of DNA lesions) are primarily repaired through the nucleotide excision repair (NER) during G1 phase of the cell cycle^1–3^. Inter-strand crosslinks (ICL) that represent less than 5% of DNA lesions, if un-repaired, can block DNA replication. ICL is processed through the Fanconi anaemia (FA) pathway during which double strand breaks (DSB) repair intermediates are generated^4–6^. During S/G2 phase of the cell cycle, DSBs are repaired through the high-fidelity homologous recombination (HR)^7^. The error-prone non-homologous end joining (NHEJ) is involved in the repair of DSBs during G1 phase^7^. The Mre11-Rad50-Nbs1 (MRN) complex is critical for DSB recognition and repair^8–10^. Mre11 nuclease is a key component of the MRN complex. Mre11, has endo- and exonuclease activities. The endonuclease activity is required during HR and 3′-5′ exonuclease activity of Mre11 contributes to the processing of stalled replication forks. Mre11 has an N-terminal nuclease domain, a RAD50 binding motif and a C-terminal DNA binding domain. Rad50 has ATP and Mre11 binding sites. Nbs1 has N-terminal BRCT & FHA domains and a C-terminal Mre11 & ATM (ataxia-telangiectasia mutated protein kinase) binding domains. The interaction of Mre11 with Rad50 and Nbs1 promotes MRN stability^8–10^. The interaction of MRN with ATM^11^ and ATR (ataxia-telangiectasia related protein kinase)^8–10^ is key to the coordination DNA repair and cell cycle progression.

Synthetic lethality is a new approach to personalize ovarian cancer therapy^12,13^. Poly-(ADP)-ribose polymerase 1 (PARP1), a key DNA repair factor binds to single-strand breaks, gets activated leading to synthesis of PAR (poly-ADP-ribose) polymers. Auto-PARylation of PARP1 promotes recruitment other DNA repair factors (including XRCC1) at sites of DNA damage to facilitate DNA repair. The BRCA1 and BRCA2 genes are essential for the repair of DSB through HR. PARP inhibitors block PARP1 catalytic activity thereby preventing auto-PARylation. As a result, not only base excision repair (BER) recruitment is impaired, but PARP1 binding to DNA intermediate also disrupts replication fork progression leading onto DSB accumulation, which if unrepaired through DDR, lead to apoptosis^12,13^. In BRCA germline deficient or platinum sensitive ovarian cancers, PARP1 inhibitor (Niraparib, Rucaparib, Olaparib, Talazoparib) maintenance therapy improves progression-free survival^12,13^. However, not all patients respond to PARP inhibitor therapy; either due to intrinsic or acquired resistance to PARP inhibitors^12,13^. Therefore, the discovery of alternative synthetic lethality approaches is a high priority in epithelial ovarian cancers.

Besides intra-strand and inter strand cross links, platinum compounds can also generate reactive oxygen species (ROS) that cause oxidative DNA base damage which is repaired through base excision repair (BER)^14^. XRCC1 (X-ray repair cross-complementing gene 1) has pivotal roles during BER and single strand break repair (SSBR)^15^. Interaction of XRCC1 with PARP1 promotes coordination of BER/SSBR^16^. XRCC1 also has roles during alternative non-homologous end joining (alt-NHEJ) pathway for double-strand breaks (DSBs) and nucleotide excision repair^15^. XRCC1 deficiency can delay SSB rejoining and lead to SSB accumulation. SSBs if unrepaired can result in the accumulation of double-strand breaks (DSBs)^15,17,18^. XRCC1 also interacts with Mre11 in response to ionizing radiation to form a microhomology-mediated end joining (MMEJ)-competent complex. During replication fork processing, XRCC1 can promote stalled fork degradation and replication restart in BRCA2-deficient cells^19,20^.

Here we have comprehensively evaluated Mre11 in ovarian cancer. We identified Mre11 as a key predictor of clinical platinum resistance. Pre-clinically, Mre11 depletion or blockade not only reversed platinum resistance, but targeting Mre11 in XRCC1 deficient platinum sensitive ovarian cancers also induced synthetic lethality.

## Results

### Mre11 expression and clinicopathological features

**Fig. 1:**
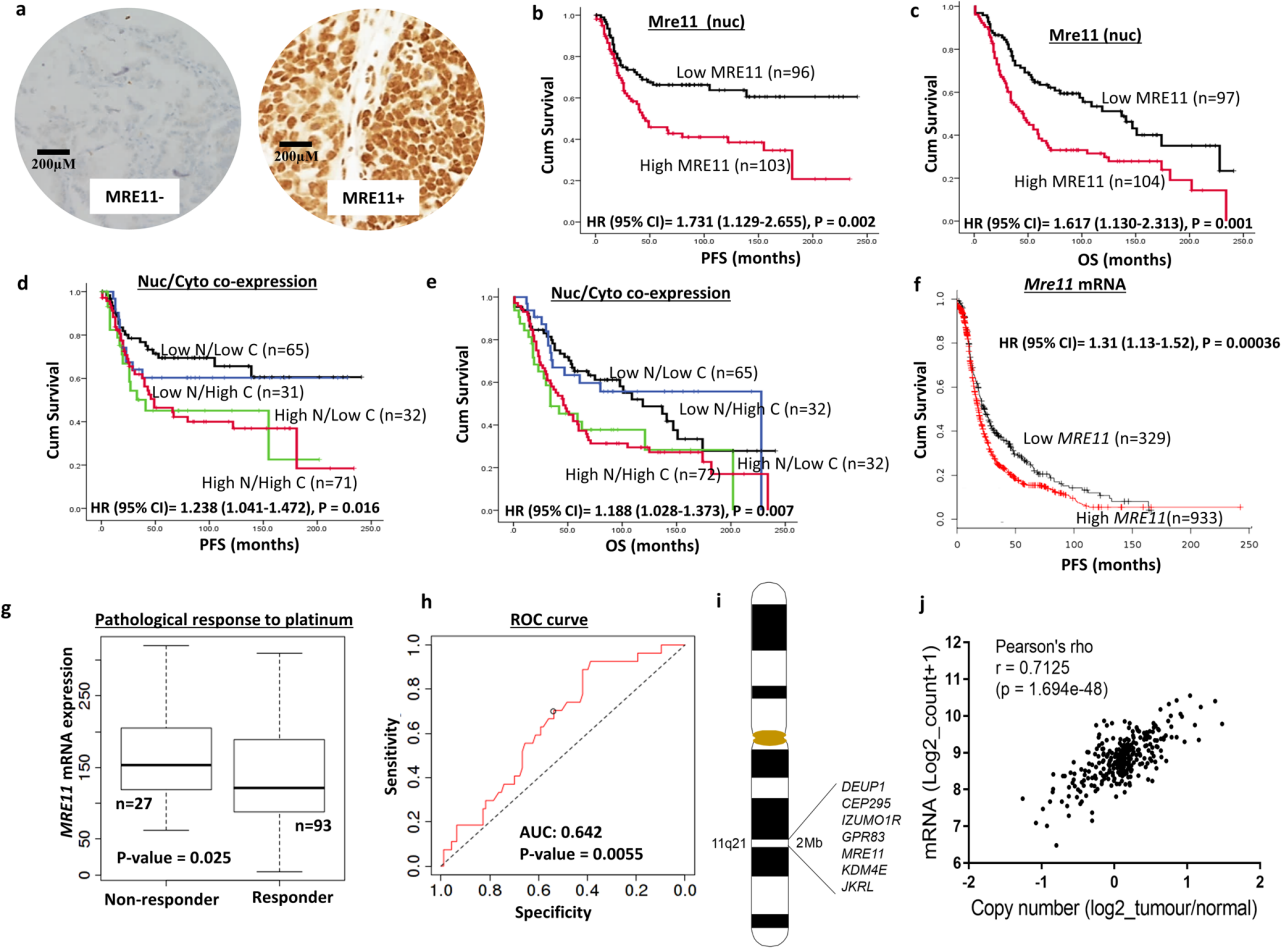
Clinicopathological studies of MRE11 expression in ovarian cancers. **a** Immunohistochemical expression of Mre11in ovarian cancers. In Mre11 positive tumour (right panel), nuclear Mre11 H-score was 280. **b** Kaplan-Meier curve for Mre11nuclear protein expression and progression free survival (PFS) in ovarian cancers (**c**) Kaplan-Meier curve for Mre11nuclear protein expression and overall survival (OS) in ovarian cancers. **d** Kaplan-Meier curve for Mre11 nuclear & cytoplasmic co-expression and progression free survival (PFS) in ovarian cancers. **e** Kaplan-Meier curve for Mre11nuclear &cytoplasmic co-expression and overall survival (OS) in ovarian cancers. **f** Kaplan Meier curve for *Mre11* mRNA expression and progression free survival (PFS) in ovarian cancers. **g** Pathological response to platinum and Mre11 mRNA expression between non responders (*n* = 27) and responders to platinum (*n* = 93). Box Plot- lower whisker [Minimum (*Q*_0_ or 0th percentile)] is the lowest data point in the data set excluding any outliers. Upper whisker [Maximum (*Q*_4_ or 100th percentile)] is the highest data point in the data set excluding any outliers. Central line [Median (*Q*_2_ or 50th percentile)]is the middle value in the data set. Bounds of the box - First quartile (*Q*_1_ or 25th percentile) is the median of the lower half of the dataset. Third quartile (*Q*_3_ or 75th percentile) is the median of the upper half of the dataset. The box is drawn from *Q*_1_ to *Q*_3_ with a horizontal line drawn in the middle to denote the median. **h** Receiver operating characteristics (ROC) for *Mre11* mRNA expression and platinum response. **i** Region of gene enrichment covers 2 Mb region of Chr11q21. **j** Correlation between RNAseq mRNA levels and copy number (*n* = 305) shows significant positive correlation *r* = 0.7125, *p* < 0.01.

Patient demographics are summarized in Supplementary Table 1. Investigation of the expression of Mre11 was carried out on tissue microarrays (TMA) of 331 consecutive ovarian epithelial cancer cases treated at Nottingham University Hospitals (NUH) between 1997 and 2010. Not all cores within the TMA were included for IHC analysis due to missing cores or absence of tumour cells. In 199 evaluable tumours, high nuclear Mre11 (Fig. 1a) was associated with serous cystadenocarcinoma (*p* < 0.001) and high FIGO stage (*p* = 0.002) **(**Supplementary Table 2**)**. High nuclear Mre11 was linked with poor progression free survival (PFS) (*p* = 0.002) **(**Fig. 1b**)** and poor overall survival (OS) (*p* = 0.001) (Fig. 1c). High cytoplasmic expression of Mre11 was associated with serous cystadenocarcinoma (*p* < 0.001) and high grade (p = 0.007) tumours **(**Supplementary Table 3**)** but did not influence survival (Supplementary Fig. 1A, B**)**. When nuclear and cytoplasmic expression of Mre11 was combined together, we observed that high nuclear/high cytoplasmic levels of Mre11 was associated with poor PFS (*p* = 0.016) **(**Fig. 1d**)** and poor OS (*p* = 0.007) **(**Fig. 1e**)** compared to tumours with low nuclear/low cytoplasmic Mre11 expression. In multivariate analyses, Mre11 (nuclear) expression remained independently associated with poor PFS (*p* = 0.019) (Supplementary Table 14) and OS (*p* = 0.029) (Supplementary Table 5). The interaction of Mre11 with Rad50 and Nbs1 promotes MRN stability. Platinum-induced oxidative base damage is repaired through base excision repair (BER). In addition, Mre11 has roles during BER/SSBR^8–10^. We have recently shown that overexpression of NBS1^21^, RAD50^22^, XRCC1^23^, polβ^24^, PARP1^25^, FEN1^26^, LIG1^27^ and LIG3^27^ predict platinum resistance in ovarian cancer. As expected, we observed a strong positive correlation between the expression of Mre11 protein and the expression of Rad50 and Nbs1 (all *ps* < 0.001) (Supplementary Table 6). Interestingly, there was also a highly significant association between Mre11 and the expression of XRCC1, polβ, FEN1, PARP1, LIG1 and LIG3 (all *ps* < 0.001) (Supplementary Table 6**)**. The data further supports the hypothesis that Mre11 either directly or indirectly through its interaction with other DNA repair factors could influence platinum resistance in ovarian cancers. At the transcriptomic level, high *MRE11* mRNA expression remained significantly associated with poor PFS (*p* = 0.00036) **(**Fig. 1f**)** and borderline non-significant for OS (*p* = 0.068) **(**Supplementary Fig. 1C**)**. Furthermore, low *Mre11* transcript was associated with pathological response to chemotherapy (*p* = 0.025) in serous cystadenocarcinomas (Fig. 1g**)**. ROC analysis revealed an area under the curve (AUC) of 0.642 (*p* = 0.0055) (Fig. 1h**)** implying potential clinical utility of *Mre11* as predictive biomarker.

### *Mre11* transcript level and genome wide alterations

Besides a role in DDR, Mre11 is essential for countering oncogene-driven replication stress. Mre11-ATM axis is also involved in pro-survival signalling, epithelial mesenchymal transition (EMT), invasion and migration^8–10^. To understand whether Mre11 dysregulation will have genome wide consequences, we conducted bioinformatics analysis in ovarian serous cystadenocarcinoma TCGA cohort^28^. Tumours were separated into quartiles depending on the level of *Mre11* mRNA. Comparison was performed between the quartile Q1 (low *Mre11*) against quartile Q4 (high *Mre11*) groups. There were 759 specimens in the TCGA RNAseq analysis, of which 605 were primary tumour, and 135 were normal. Of the primary tumours, 325 were low Mre11, and 90 were high MRE11 (significantly lower in Q1 vs Q4 log2FC = −1.283840657). For non-malignant samples, 135 of 135 were low Mre11. Differential gene analysis identified 867 transcripts expressed higher in Q1 (low *Mre11*) and 692 transcripts higher in Q4 (high *Mre11*) (Supplementary Data 1). We interrogated a selected set of previously described markers of platinum resistance in TCGA cohort. As expected, SHH (log2FC = −1.958209627), FGF4 (log2FC = −1.847099153), FGF20 (log2FC = −1.744137973), FGF19 (log2FC = −1.711349003), FGF 17 (log2FC = −1.682894019) and FGF9 (log2FC = −1.091264547 were significantly lower in Q1 compared to Q4 **(**Supplementary Fig. 1D**)**. Pathway analysis of the differential genes identified 5 significant pathways for genes expressed in low *MRE11* group (Supplementary Data 2 & Supplementary Table 7). The genes that represented the pathways were predominately the glutathione S-transferase Alpha 1/2 (*GSTA1, GSTA2*). Glutathione metabolism has been shown to be involved in cisplatin resistance, by sequestering cisplatin which in turn prevents cisplatin binding DNA and DNA damage^29^. No significant pathways were identified in the genes showing higher expression with high *Mre11*. We did identify genes in the high *MRE11* group that have been shown to play a role in platinum-based resistance in ovarian cancer. These included are the FGFs and FGF receptors^30^, SHH^31^ and Progesterone receptor^32^.

Interestingly, chr11q21 was significantly highlighted as chromosomal location for gene enrichment (FDR 2.43E-05). Differential genes (logFC > 2; FDR < 0.05) in this 2 Mb region included *Mre11, CP295, DEUP1, IZUMO1R, JRKL* and *KDM4E* (Fig. 1i). *DEUP1* has been shown to be silenced by DNA methylation in liver and gastric cancers^33,34^. Both *IZUMO1R* and *KDM4E* have been shown to play a role in oocyte, fertilisation and embryogenesis, but very little is known about their role in cancer^35,36^. We identified significant positive correlation between Mre11 copy number and mRNA levels (Pearson correlation *r* = 0.71, *p* < 0.01; Fig. 1j). For the cBioportal analysis, GISTIC 2.0 putative gene copy number analysis was utilized on the TCGA dataset within cBIOportal. Gain is stated as having a few additional copies, whereas amplification is greater than this, so identified as many copies. Interestingly, no mutations within the MRE11 gene were identified in the 585 samples (TCGA Pan Cancer cohort). We identified copy number gains in Mre11 and in 5% cases (10/201 tumours) amplification (Supplementary Fig. 2A). The genes within 11q21 showed amplification within the same tumours and alterations in chr11 have been previously shown, but not investigated further at 11q due to the lower frequency of alterations compared to other chromosomal aberrations^28,37^. Additionally, utilising the TCGA Ovarian cancer cohort in UCSC Xena, we observed a non-significant trend (Supplementary Fig. 2B, *p* = 0.08) toward worse survival in patients with tumours that had high Mre11 copy numbers (Supplementary Fig. 2B).

Taken together, the data provides clinical evidence high Mre11 may predict adverse clinical outcome and resistance to platinum therapy in ovarian cancer patients. To validate this further we proceeded to pre-clinical evaluation in ovarian cancer cell lines.

### *Mre11* gene sequencing, protein expression and sub cellular localization in platinum sensitive and resistant ovarian cancer cell lines

**Fig. 2:**
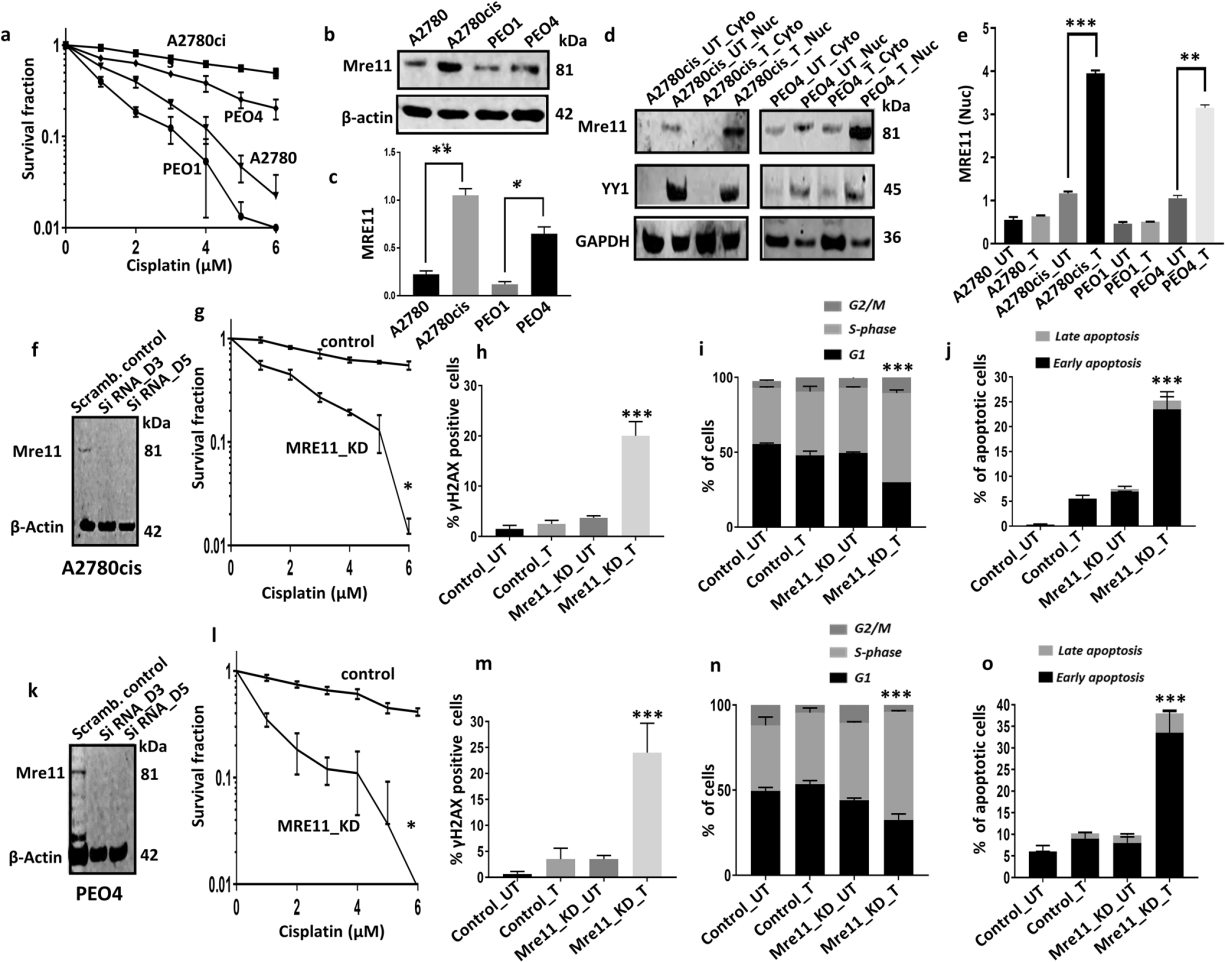
MRE11 depletion and cisplatin sensitivity in ovarian cancer cells. **a** Clonogenic survival assay for cisplatin sensitivity in A2780, A2780cis, PEO1 and PEO4 cell lines. **b** Western blot showing MRE11 protein levels in A2780, A2780cis, PEO1 and PEO4 ovarian cell lines. **c** Quantification of Mre11 protein levels by western blot in A2780, A2780cis, PEO1 and PEO4 cell lines. Normalization to β-Actin for the quantification of the Mre11 in western blot bands were performed. **d** Mre11 levels in nuclear and cytoplasmic extracts of A2780cis and PEO4 treated with 5 µM cisplatin. Lysates were collected 24 h post treatment (UT = untreated with cisplatin, T = treated with cisplatin). **e** Mre11 protein levels in nuclear extracts of A2780, A2780cis, PEO1 and PEO4 cells treated with 5 µM cisplatin for 24 h. **f** Mre11 siRNA knock down in A2780cis cells. Lysates were collected at day3 and day5. **g** Clonogenic survival assay for cisplatin sensitivity in A2780cis cells control and A2780cis_MRE11_KD cells. **h** Quantification of γH2AX positive cells by flow cytometry. **i** Cell cycle analysis by flow cytometry. **j** AnnexinV analysis for apoptotic cells in A2780cis control and MRE11_knock down cells treated with 5 μM cisplatin. **k** Mre11 siRNA knock down in PEO4 cells. **l** Clonogenic survival assay for cisplatin sensitivity PEO4 cells control and PEO4_Mre11_KD cells. **m** Quantification of γH2AX positive cells by flow cytometry. **n** Cell cycle analysis by flow cytometry. **o** AnnexinV analysis for apoptotic cells in PEO4 control and Mre11_knock down cells treated with 5 μM cisplatin. Figures are representative of three or more independent experiments. Error bars represent standard error of mean between experiments. The *P* value was calculated as follow, *p > 0.05, **p > 0.01 and ***p > 0.001. All western blots were derived from the same experiment and were processed in parallel.

We evaluated a panel of ovarian cancer cell lines. Platinum sensitive A2780 cell line was established from a patient with previously untreated ovarian cancer. Platinum resistant A2780cis cell line was developed by chronic exposure of the parental A2780 cell line to increasing concentrations of cisplatin. Platinum sensitive PEO1 cell line was derived from a patient with a poorly differentiated serous adenocarcinoma. Platinum resistant PEO4 cell line was established from the same patient after the development of clinical resistance to platinum chemotherapy. In clonogenic assays, we first confirmed platinum resistance in A2780cis and PEO4 cells compared to A2780 and PEO1 cells (Fig. 2a).

Germ-line mutations in Mre11 causes ataxia telangiectasia-like disorder (ATLD), a genomic instability syndrome characterised by immunodeficiency, genomic instability, hypersensitivity to radiation and cancer predisposition. Polymorphic variation in *Mre11* may also be associated with increased cancer predisposition. We performed next generation exome sequencing (NGS) in A2780, A2780cis, PEO1 and PEO4 cell lines. The full NGS data has been uploaded and is available at NCBI-GEO (https://www.ncbi.nlm.nih.gov/geo/) with the following accession numbers GSE198648 (PEO1, PEO1R cell lines) and GSE160540 (PEO1, PEO1R xenografts). Deep sequencing did not reveal any coding variants of *Mre11* gene in these cell lines. One known intronic variant, rs35062043, which does not affect the coding sequence was identified in A2780cis cells which was absent in the parental A2780 cell line. No variants in Mre11 were identified in PE01 or PE04 cells. A summary of coding variants identified in high confidence components of the Mre11 interactome in A2780cis cells is shown in Supplementary Table 8. Key interactors included Rad50, Nbs1 and ATM, all previously known to interact with Mre11^8–10^.

In whole cell lysates, baseline Mre11 protein level was high in platinum resistant A2780cis and PEO4 cells compared to platinum sensitive A2780 and PEO1 cells respectively (Fig. 2b and c**)**. To monitor Mre11 sub-cellular localization, we generated nuclear and cytoplasmic extracts following 24 h of cisplatin therapy (Fig. 2d, Supplementary Fig. 3A, B). In A2780cis and PEO4 cells, platinum treatment increased nuclear localisation of Mre11 compared to A2780 and PEO1 cells (Fig. 2e). No significant alterations were observed for cytoplasmic expression of Mre11 in A2780, A2780cis, PEO1 and PEO4 cells (Fig. 2d, Supplementary Fig. 3A–C). In a panel of breast cancer lines (MCF7, MDA-MB-231, MDA-MB-436, SKBR-3, MDA-MB-175VII), we also evaluated Mre11 at baseline and after cisplatin treatment (Supplementary Fig. 3D and E**)**. Whereas MCF7, MDA-MB-231 and MDA-MB-436 had basal levels of Mre11 expression, SKBR-3 and MDA-MB-175VII cells were negative for Mre11 expression (Supplementary Fig. 3D**)**. Cisplatin treatment did not significantly increase Mre11 levels in MCF7, MDA-MB-436, SKBR-3 and MDA-MB-175VII cells. Interestingly, Mre11 level decreased in MDA-MB-231 cells after cisplatin treatment (Supplementary Fig. 3D**)**. Taken together, the data suggest that increase in Mre11 levels after cisplatin treatment is a feature of ovarian cancer cell lines.

In co-immunoprecipitation experiments, in ovarian cancer cell lines, we also observed that Mre11 physically interacted with Nbs1, Rad50, XRCC1 and LIG3 (Supplementary Fig. 3F**)** consistently in PEO1 cells. We have recently shown that overexpression of Nbs1^21^, Rad50^22^, XRCC1^23^ and LIG3^27^ predict platinum resistance in ovarian cancer. Together, the data suggests that Mre11 nuclear accumulation following platinum therapy as well as Mre11 interaction with XRCC1 and LIG3 could promote repair of platinum induced DNA damage and contribute to resistance. To explore this possibility further, we depleted Mre11 in platinum resistant A2780cis and PEO4 cells and investigated for platinum re-sensitization.

### Mre11 depletion reverses platinum resistance

**Fig. 3:**
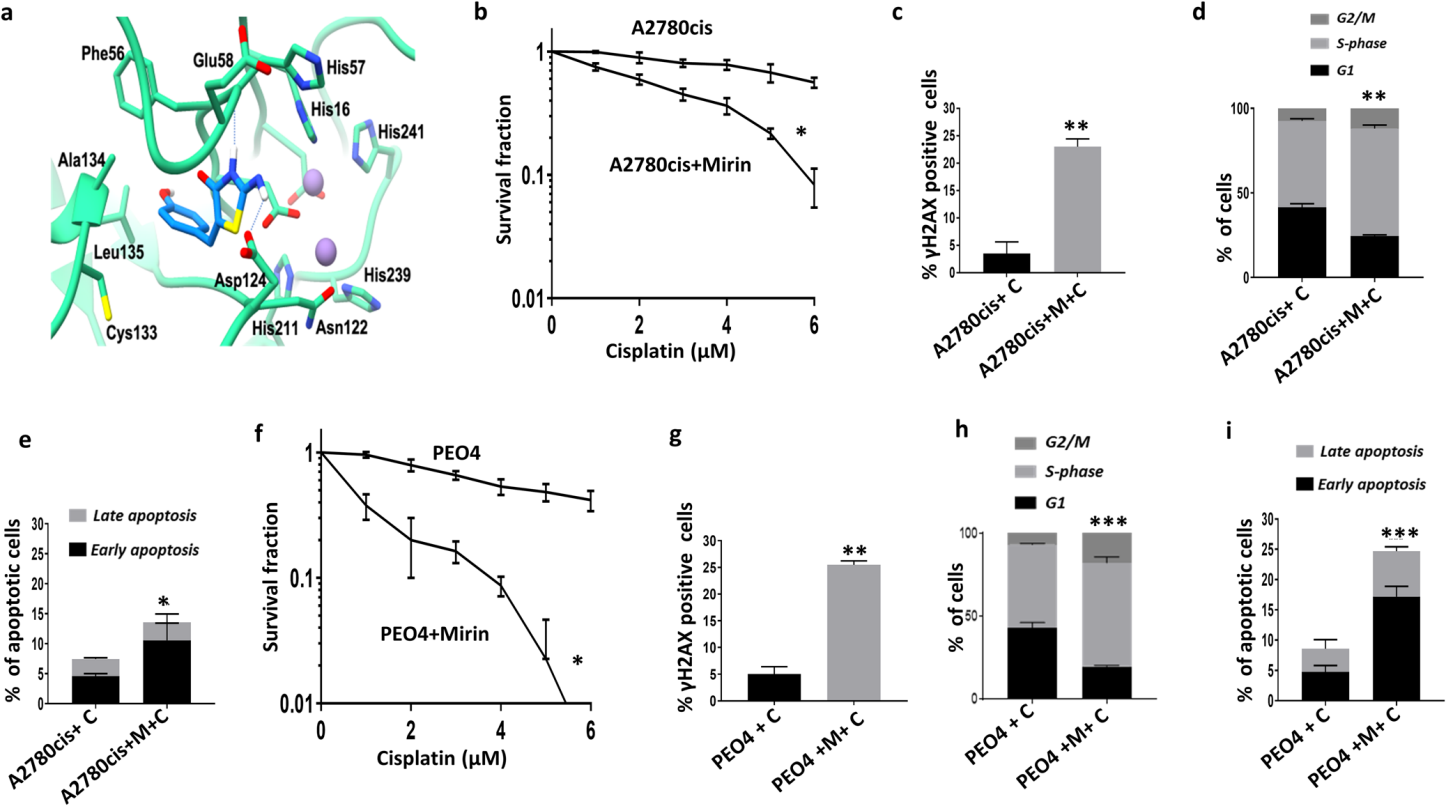
MRE11 blockade with Mirin and cisplatin sensitivity in ovarian cancer cells. **a** Binding pose of Mirin (blue) to Mre11 (green) obtained by molecular docking, suggesting Mirin forms a hydrogen bond to the backbone of Glu58 (dashed line). **b** Clonogenic survival assay for A2780cis cells untreated and pre -treated with Mirin. **c** γH2AX positive cells by flow cytometry in A2780cis cells treated with cisplatin alone or treated with Mirin plus cisplatin. **d** Cell cycle analysis by flow cytometry in A2780cis cells treated with cisplatin alone or treated with Mirin plus cisplatin. **e** AnnexinV analysis for apoptotic cells in A2780cis cells treated with cisplatin alone or treated with Mirin plus cisplatin. Cells were plated overnight then treated with Mirin (10 μM) for 24 h. The following day cells were treated with 5 μM cisplatin for another 24 h, then collected for cell cycle analysis or AnnexinV analysis by flow cytometry. **f** Clonogenic survival assay for PEO4 cells untreated and pre-treated with Mirin. **g** γH2AX positive cells by flow cytometry in PEO4 cells treated with cisplatin alone or treated with Mirin plus cisplatin. **h** Cell cycle analysis by flow cytometry. **i** AnnexinV analysis for apoptotic cells in PEO4 cells treated with cisplatin alone or treated with Mirin plus cisplatin. Figures are representative of three or more independent experiments. Error bars represent standard error of mean between experiments. The *P* value was calculated as follow, *p > 0.05, **p > 0.01 and ***p > 0.001.

We first generated transient knockdowns (KD) of Mre11 using siRNAs in A2780cis (Fig. 2f). In clonogenic assays, Mre11_KD_A2780cis cells (Fig. 3g**)** were significantly sensitive to platinum compared to scrambled control. Increased cytotoxicity was associated with DSB accumulation (Fig. 2h, Supplementary Fig. 4A), S-phase cell cycle arrest **(**Fig. 2I, Supplementary Fig. 3B**)** and increased apoptosis (Fig. 2j, Supplementary Fig. 4C) compared to scrambled controls. We then depleted Mre11 in PEO4 cells **(**Fig. 2k**)**. As shown in Fig. 2l, Mre11_KD_PEO4 cells showed increased platinum sensitivity compared to scrambled controls. Increased sensitivity to cisplatin in Mre11_KD_PEO4 cells was also associated with DSB accumulation (Fig. 2m), S-phase arrest (Fig. 2n) and increased apoptotic cells (Fig. 2o). We further validated using a second siRNA construct for Mre11 depletion (Supplementary Fig. 4D) and confirmed platinum sensitization in Mre11_KD_A2780cis cells (Supplementary Fig. 4E**)** compared to scrambled control.

### Mre11 blockade by small molecule inhibitor increases platinum sensitivity

Mirin [Z-5-(4- hydroxybenzylidene)-2-imino-1,3-thiazolidin-4-one] is a small molecular inhibitor of the exonuclease activity of Mre11^38^. We initially conducted structural bioinformatics studies using previously published crystal structures of Mre11 to understand the docking of Mirin onto the active site of Mre11 (Fig. 3a). Structure of human Mre11 (PDB: 3t1i, PMID 20122942) was prepared for docking by removal of solvent (DTT, glycerol and water) and superposed to the TmMre11:Mirin complex (PDB: 4o4k, PMID 24316220). Owing to differences in the two homologues, the position of loop formed by residues HsMre11 loop of residues 127–131, of which Asn127 co-ordinates one of the bound catalytic manganese ions, precludes mirin binding without conformational change. Despite its conformation close to the catalytic ions in the *apo* structure, His129 of HsMre11 does not coordinate either manganese, suggesting by conformational change in the loop, mirin could bind to inhibit Mre11 catalytic activity. To this end, residues 129–132 were removed from the HsMre11 structure and re-modelled using MODELLER via UCSF Chimera. A solution was selected which best matched the TmMre11:mirin complex conformation of loop 93-93. Induced-fit docking of mirin to the HsMre11 model was performed in MOE 2018.01 based on the position of mirin in the TmMre11 complex. Initial placement used triangle matching for 30 poses using London dG scoring; for refinement, protein side chains were freely flexible and poses were scored using Affinity dG; ten poses were analysed. The top scoring pose was in best agreement with the TmMre11:Mirin complex structure, though shows mirin able to adopt an alternate conformation to form hydrogen bonds with the backbone of Glu58 and the side-chain of Asp124 while positioning the phenol moiety into a hydrophobic pocket formed by residues including Phe56 and Leu135 (Fig. 3a).

We then conducted cisplatin chemo-potentiation studies in A2780cis and PEO4 cells. Mirin pre-treatment for 24 h increased sensitivity to platinum in A2780cis cells (Fig. 3b**)**. Increased sensitivity was associated with DSB accumulation (Fig. 3c**)**, S-phase arrest (Fig. 3d**)** and increased apoptotic cells (Fig. 3e**)**. Similarly, in PEO4 cells, Mirin pre-treatment increased platinum sensitivity (Fig. 3f**)**, increased DSB accumulation (Fig. 3g**)**, S-phase arrest (Fig. 3h**)** and apoptotic cells (Fig. 3i**)**. These data provide evidence that Mre11 depletion or blockade is not only a platinum sensitizer, but Mre11 is also an attractive anti-cancer target. We then proceeded to investigate if Mre11 blockade can be exploited for novel synthetic lethality applications.

### Mre11 inhibition is synthetically lethal in XRCC1-deficient cancer cells

XRCC1 is a key player in BER, SSBR, NER and alt-NHEJ DNA repair pathways^15^. XRCC1 also has a role in the maintenance of replication fork stability^15^. XRCC1 has been shown to interact with Mre11^20^. We have previously shown that XRCC1 deficiency is a predictor of platinum sensitivity in ovarian cancers^23^. Our hypothesis is that XRCC1 deficiency will lead to SSB accumulation which will get converted to DSBs during replication. In addition, XRCC1 deficiency will also lead to replication fork instability and DSB formation. Mre11 inhibition will block DSB initiated DDR thereby promoting accumulation of lethal DSBs and cell death.

**Fig. 4:**
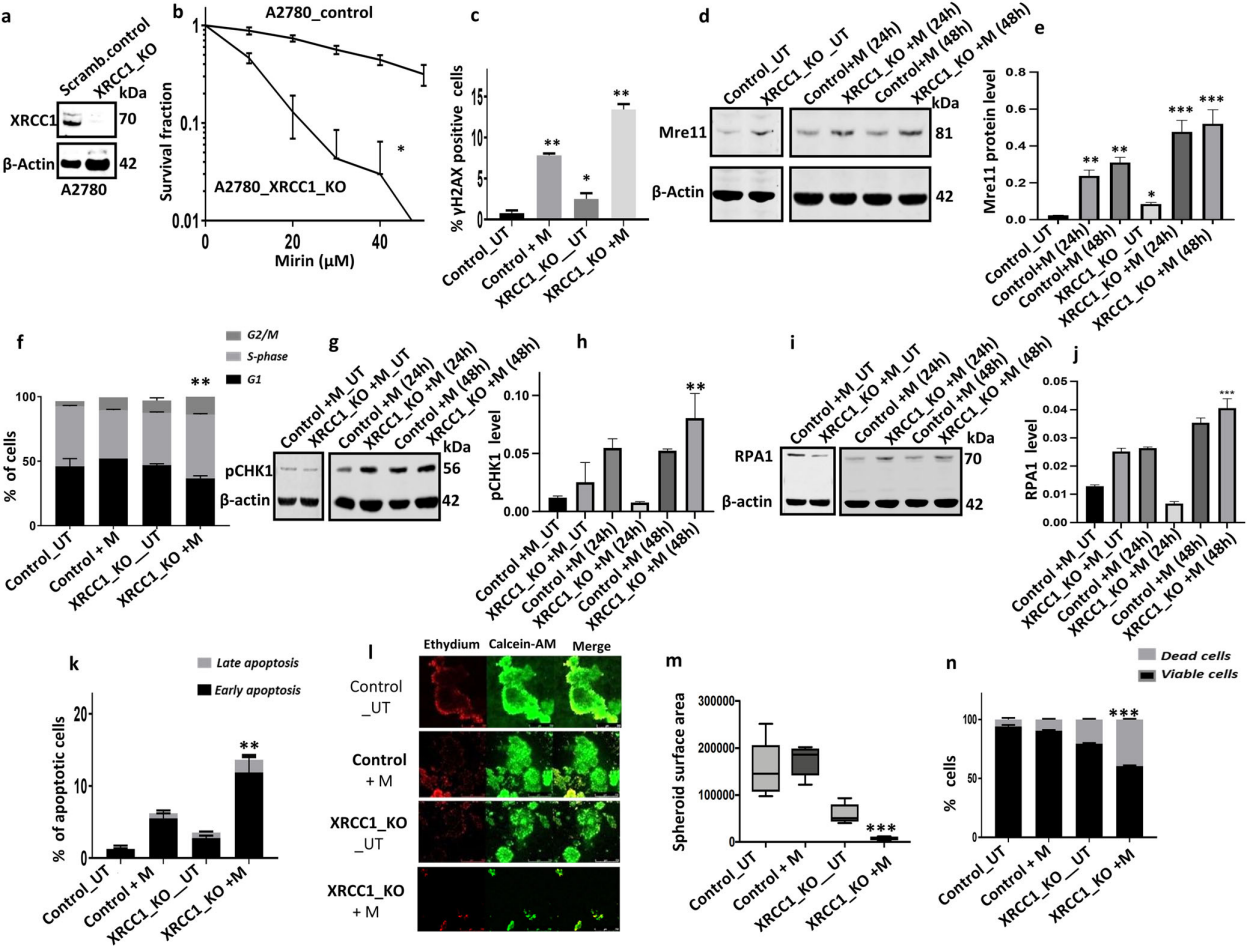
Mirin induced synthetic lethality in XRCC1 deficient ovarian cancer cells. **a** XRCC1knock out by CRISPR-cas9 in A2780 cells. **b** Clonogenic survival assay for Mirin sensitivity in A2780 control and A2780_XRCC1_KO cells. **c** γH2AX positive cells by flow cytometry in A2780 control and A2780_XRCC1_KO cells untreated or treated with Mirin (25 μM) for 24 h. **d** Western blot of Mre11 expression in A2780_XRCC1_KO cells untreated or treated with Mirin (25 μM) for 24 h and 48 h. **e**. Mre11 protein quantification in A2780_XRCC1_KO cells untreated or treated with Mirin (25 μM) for 24 h and 48 h. **f** Cell cycle analysis by flow cytometry in A2780_XRCC1_KO cells untreated or treated with Mirin (25 μM) for 24 h. **g** Western blot of pChk1 expression in A2780_XRCC1_KO cells untreated or treated with Mirin (25 μM) for 24 h & 48 h. The western blot shown for untreated and treated cells were from the same experiment. **h** pChk1 protein quantification in A2780_XRCC1_KO cells untreated or treated with Mirin (25 μM) for 24 h and 48 h. Normalization to β-Actin for the quantification of the pChk1 was performed. **i** Western blot of RPA1 expression in A2780_XRCC1_KO cells untreated or treated with Mirin (25 μM) for 24 h and 48 h. The western blot shown for untreated and treated cells were from the same experiment. **j** RPA1 protein quantification in A2780_XRCC1_KO cells untreated or treated with Mirin (25 μM) for 24 h and 48 h. Normalization to β-Actin for the quantification of the pChk1 was performed. **k** AnnexinV analysis for apoptotic cells in A2780 control and A2780_XRCC1_KO cells untreated or treated with Mirin (25 μM) for 24 h. **l** Representative photomicrographic images for 3D spheroids of A2780 control and XRCC1_KO cells treated with Mirin (25 μM) for 48 h. Images showing magnification x20. **m** Quantification of spheroids size by ImageJ software. **n** Quantification of spheroids cell viability by flow cytometry. Figures are representative of three or more independent experiments. Error bars represent standard error of mean between experiments. The P value was calculated as follow, *p > 0.05, **p > 0.01 and ***p > 0.001. All western blots were derived from the same experiment and were processed in parallel.

**Fig. 5:**
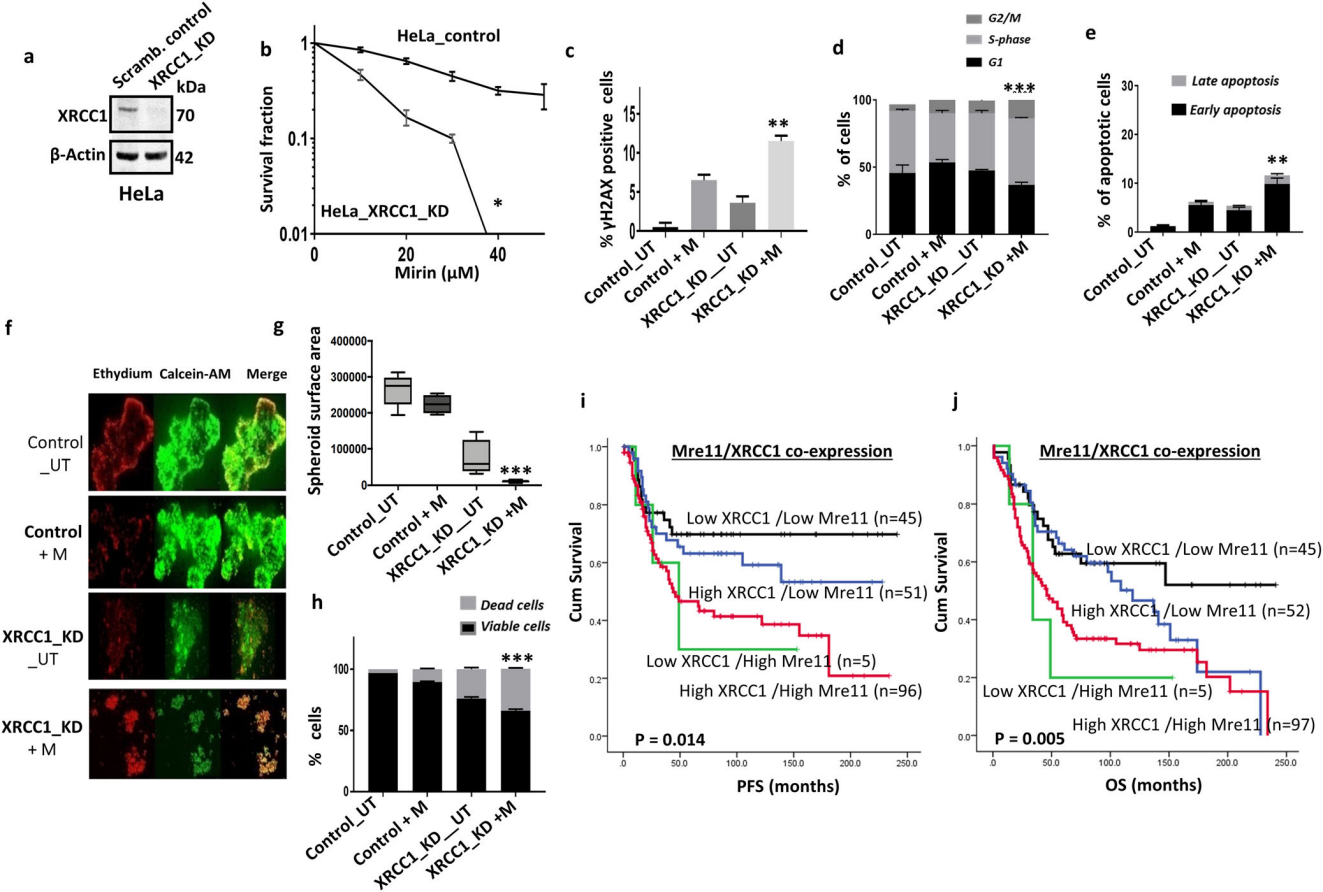
Mirin induced synthetic lethality in XRCC1 deficient HeLa cancer cells. **a** XRCC1 knock down by sh-RNA in HeLa cells. **b** Clonogenic survival assay for Mirin sensitivity in HeLa control and HeLa_XRCC1_knock down cells. **c** γH2AX positive cells by flow cytometry. **d** Cell cycle analysis by flow cytometry. **e** AnnexinV analysis for apoptotic cells in HeLa control and HeLa_XRCC1_knock down cells treated with 25 μM Mirin for 24 h. **f** Representative photomicrographic images for 3D spheroids of HeLa control and HeLa_XRCC1_knock down cells treated with Mirin (25 μM) for 48 h. Images showing magnification x20. **g** Quantification of spheroids size by ImageJ software. **h** Quantification of spheroids cell viability by flow cytometry. **i** Kaplan-Meier curve for Mre11&XRCC1 co-expression and progression free survival (PFS) in ovarian cancers. **j** Kaplan-Meier curve for Mre11&XRCC1 co-expression and overall survival (OS) in ovarian cancers. Figures are representative of three or more independent experiments. Error bars represent standard error of mean between experiments. The P value was calculated as follow, *p > 0.05, **p > 0.01 and ***p > 0.001.

We therefore tested for Mirin induced synthetic lethality in XRCC1 deficient cells. We have recently generated XRCC1_KO ovarian cancer cells using CRISPR/Cas-9 methodology in A2780 cells^25^**(**Fig. 4a**)**. We evaluated Mirin sensitivity in XRCC1_KO cells and compared to scrambled control cells. As shown in Fig. 4b, A2780_XRCC1_KO cells were sensitive to Mirin treatment compared to control cells (IC_50_ = 20 µM). Although Mirin treatment in control cells increased γH2AX positive cells, this was significantly further increased in A2780_XRCC1_KO cells compared to control cells (Fig. 4c). We then tested Mre11 levels in whole cell extracts from untreated samples, after 24 h, and 48 h of Mirin treatment in XRCC1_KO cells compared to scrambled controls (Fig. 4d, e). Basal Mre11 levels was increased in XRCC1_KO cells compared to scrambled controls (Fig. 4d and e). Mirin treatment further increased Mre11 levels at 24 h and 48 h in XRCC1_KO cells compared to scrambled controls (Fig. 4d, e). Increased Mre11 expression upon Mirin treatment is likely due to accumulation of DSBs which induce Mre11 expression as a feedback loop. Increased DSB was associated with S-phase arrest in Mirin treated XRCC1_KO cells compared to scrambled controls (Fig. 4F). S-phase arrest was associated with increased pCHK1 (Fig. 4g, H), increased replication protein A1 (RPA1) (Fig. 4I, J) levels and accumulation of apoptotic cells (Fig. 4k) in Mirin treated XRCC1_KO cells compared to controls. To recapitulate an in vivo system, we also generated 3D-spheroids of A2780_control cells and A2780_XRCC1_KO cells. Compared to A2780_control spheroids, A2780_XRCC1_KO spheroids were reduced in size at baseline (Fig. 4l, m**)**. Mirin treatment reduced size further in XRCC1-deficient A2780 spheroids (Fig. 4l, m**)** and lead to accumulation of dead cells compared to control spheroids (Fig. 4n**)**. For further validation we also tested HeLa control and HeLa _XRCC1-knockdown (KD) cells (Fig. 5a). As expected, HeLa_XRCC1_KD cells were extremely sensitive to Mirin treatment (Fig. 5b) compared to controls which was associated with DSB accumulation (Fig. 5c), S-phase arrest (Fig. 5d) and apoptosis (Fig. 5e). At baseline, HeLa_XRCC1 deficient spheroids were smaller compared to control spheroids (Fig. 5f, g**)**. Upon Mirin therapy, spheroid substantially reduced in size (Fig. 5f, g**)** with accumulation of dead cells (Fig. 5h**)**.

We have previously shown that low XRCC1 protein expression (seen in about 33% of epithelial ovarian cancers) is associated with platinum sensitivity^23,25^. Here, we evaluated if Mre11/XRCC1 co-expression would also influence clinical outcomes. As expected, patients whose tumours had low XRCC1/low Mre11 co-expression (22.8%) had better progression free survival (*p* = 0.014, Fig. 5I) and overall survival (*p* = 0.005, Fig. 5j) compared to tumours that had high XRCC1/high Mre11 co-expression (48.7%).

The data in its entirety therefore provides compelling clinical and pre-clinical evidence that targeting Mre11 is an attractive anti-cancer strategy in ovarian cancers.

## Discussion

Mre11 nuclease, a critical player during DDR, is required for HR and processing of stalled replication forks^8–10^. Here we have comprehensively evaluated Mre11 in ovarian cancer. We show that Mre11 is a predictor of clinical platinum resistance. Pre-clinically, Mre11 depletion or blockade not only reversed platinum resistance, but we also identified a synthetic lethality approach targeting Mre11 in XRCC1 deficient platinum sensitive ovarian cancers. Taken together, the data provides evidence that Mre11 is an attractive drug target in epithelial ovarian cancer. Mre11 operates upstream of ATM and ATR network. Whilst small molecule inhibitors of ATM and ATR are under clinical trial evaluation, Mre11 blockade could impair both ATM and ATR mediated pathways simultaneously and could be an effective strategy in ovarian cancers which frequently manifest genomic instability and replication stress.

At the transcriptomic and protein levels Mre11 overexpression was linked with aggressive phenotypes and poor PFS in patients who received platinum-based chemotherapy. Whilst nuclear Mre11 may lead to increased DNA repair capacity and promote platinum resistance, we also observed cytoplasmic staining for MRE11 in ovarian tumours. Although the mechanisms of altered sub-cellular localization of Mre11 observed here is unknown, it is possible that cytoplasmic staining may indicate mitochondrial localization. Mre11 has previously been shown to translocate to mitochondria following reactive oxygen stress (ROS) induced mitochondrial DNA damage. Platinating agents, besides inducing ROS, can also directly damage mitochondrial DNA^39^. Emerging evidence also indicates a role for mitochondrial homeostasis in promoting cisplatin resistance^40,41^. In the current study, 72/201 (36%) tumors had nuclear and cytoplasmic overexpression indicating that nuclear and mitochondrial DNA damage could require Mre11. However, further mechanistic studies will be required to confirm the clinical implication of mitochondrial Mre11 in cancer cells. We have recently shown that the Mre11 partners, Rad50^22^ and Nbs1^21^ are also predictors of platinum resistance in ovarian cancers. Here, we have demonstrated a strong positive correlation between Mre11, Rad50 and Nbs1 in clinical ovarian cancer cohorts. Taken together, our data confirm a role for MRN complex in driving platinum resistance in ovarian cancers. In rectal cancers, overexpression of MRN was previously correlated with poor response to neoadjuvant radiotherapy and survival^42^. In gastric cancer, high MRN is also linked with poor response to chemotherapy^43^. In the current study, high Mre11 expression was linked with serous cystadenocarcinoma, high-stage, and high-grade disease. Interestingly in another study, low MRE11 level was more frequent in low grade ovarian tumours^44^. In the TCGA cohort, we observed a subset of tumours with Mre11 amplification which was strongly correlated with high *Mre11* mRNA expression. *Mre11* mRNA overexpression also linked with poor PFS. Our data suggest that the mechanism of Mre11 overexpression in ovarian cancer is multifactorial; gene amplification, transcriptional upregulation, protein overexpression and altered sub-cellular localization may all contribute to Mre11 upregulation and platinum resistance in tumours.

Pre-clinically, we initially deep sequenced platinum sensitive (PEO1, A2780) and resistant (PEO4, A2780cis) ovarian cancer cell lines. We did not observe any coding variants of *Mre11* gene in these cell lines. However, one limitation of the study is that we did not perform RNA seq analysis which could reveal mechanisms of overexpression of MRE11 in platinum resistant PEO4 and A2780cis cells. Interestingly, however, Mre11 depletion reversed resistance in platinum resistant ovarian cancer cell lines. A small molecular inhibitor of Mre11 (Mirin) increased platinum sensitization. Mirin can block MRN-dependent activation of ATM without affecting ATM kinase activity^38^. Mirin has been shown previously to abolish the G2/M checkpoint and homology-dependent repair in cells^38^. In the current study, when platinum resistant A2780cis and PEO4 cells were pre-treated with Mirin, we observed significant reversal of platinum resistance which was associated with DSB accumulation, S-phase arrest and increased apoptotic cells. A model for platinum sensitization is suggested as follows: Platinum induced inter-strand crosslinks are repaired through Fanconi anaemia (FA) pathway. DSB intermediates are generated during FA repair. Mre11 is critical for DSB recognition and subsequent repair. Mre11 blockade in this context will lead to accumulation of toxic DSB and cell death.

Next, we explored if Mre11 blockade could also be exploited for synthetic lethality in platinum sensitive ovarian cancers. XRCC1 is a multifunctional scaffold protein with recognized roles during BER, SSBR, NER and alt-NHEJ DNA repair pathways^15^. XRCC1 interacts with PARP1 during BER and SSBR^15^. XRCC1 is also involved in the repair of certain un-resected stalled replication forks^15^. PARP1 mediated MRE11 recruitment was shown in stalled replication forks that require XRCC1 mediated repair^45^. We have previously shown that XRCC1 deficiency is a predictor of platinum sensitivity in ovarian cancers^23^. XRCC1 deficient cells are extremely sensitive to cisplatin cytotoxicity with an IC_50_ of 0.45 µM in that study^23^. Mre11 also has recognised roles during base excision repair^46^. Here we have shown that Mre11 physically links with XRCC1 in co-IP experiments. Therefore, given the emerging links, we speculated a synthetic lethality interaction between Mre11 and XRCC1. Model for synthetic lethality is proposed as follows: XRCC1 deficiency will lead to SSB accumulation, which if unrepaired, will get converted to DSBs during replication. Moreover, XRCC1 deficiency will also contribute to replication fork instability, thereby promoting DSB accumulation. Blockade of DDR/DSB repair by Mre11 inhibition will result in accumulation of lethal DSBs leading to cell death. Accordingly, we observed selective toxicity of Mirin in XRCC1 deficient cancer cells with an IC_50_ of 20 µM. Increased cytotoxicity was associated with DSB accumulation, G2/M cell cycle arrest and accumulation of apoptotic cells. We speculate that the development more potent Mre11 inhibitor could increase the selective toxicity of Mre11 blockade in XRCC1 deficient cells. A further limitation of the current study is the lack of in vivo xenograft study validation. Whilst the data presented here is a proof-of-concept investigation, the availability of more potent future pharmaceutical inhibitors of Mre11 will allow validation in future xenograft studies.

Recently, Mre11 deficiency has also been exploited for synthetic lethality. Mre11 deficient endometrial cancer^47^ and colorectal cancer^48^ cells have been shown to be sensitive to PARP inhibitors. Our data also indicates that Mre11 deficient epithelial ovarian cancers may also be suitable for PARP targeted synthetic lethality approach.

In conclusion, Mre11 deficiency is a predictor of platinum sensitivity. Mre11 blockade is a platinum sensitizer and can induce synthetic lethality in XRCC1 deficient-platinum sensitive ovarian cancers. Pharmaceutical development of Mre11 inhibitors is a viable clinical strategy for precision oncology approaches in ovarian cancers.

## Methods

### Mre11 expression level in ovarian cancers

Investigation of the expression of Mre11 was carried out on tissue microarrays of 331 consecutive ovarian epithelial cancer cases treated at Nottingham University Hospitals (NUH) between 1997 and 2010. This study was carried out in accordance with the declaration of The Helsinki and ethical approval which was obtained from the Nottingham Research Ethics Committee (REC Approval Number 06/Q240/153). All patients provided written informed consent. The characteristics of this cohort are summarized in Supplementary Table 1.

### Tissue microarray (TMA) and immunohistochemistry (IHC)

Tumours were arrayed in tissue microarrays (TMAs) constructed with 2 replicate 0.6 mm cores from the tumours as described previously^49^. Immunohistochemical staining was performed using the Thermo Fisher Scientific Shandon Sequenza chamber system (REF: 72110017), along with the Novolink Max Polymer Detection System (RE7280-K: 1250 tests) and the Leica Bond Primary Antibody Diluent (AR9352) as per the manufacturer’s instructions (Leica Microsystems). The tissue micro array slides were deparaffinised with xylene, rehydrated through five decreasing concentrations of alcohol (100, 90, 70, 50 and 30%) for two minutes each. Antigen retrieval prior to staining was performed using sodium citrate buffer (pH 6.0) and heated for 20 min at 95 °C in a microwave (Whirlpool JT359 Jet Chef 1000 W). Slides were incubated with the primary anti-Mre11 mouse monoclonal antibody (clone ab214, Abcam), at a dilution of 1:800, for 1 h at room temperature. We have recently reported the clinicopathological significance of the expression of NBS1^21^, RAD50^22^, XRCC1^25^, polβ^24^, PARP1^25^, FEN1^26^, LIG1^27^ and LIG3^27^ in ovarian cancers. For XRCC1 staining^25^, a set of TMA slides were incubated for 15 min at room temperature with 1:200 anti-XRCC1 mouse monoclonal antibody (Ab-1, clone 33-2-5, Thermoscientific, Fremont, CA). For polβ staining^24^, the TMA sections were incubated for 60 min at room temperature with 1:200 dilutions of anti-pol β rabbit polyclonal antibody (clone ab26343, Abcam). For NBS1 staining^21^, A set of slides were incubated with the primary anti-NBS1 rabbit monoclonal antibody (N3162, Sigma), at a dilution of 1:200, for 60 min at room temperature. For RAD50 staining^22^, a set of slides were incubated with the primary anti-RAD50 mouse monoclonal antibody (clone ab89, Abcam), at a dilution of 1:400, for 1 h at room temperature. For LIG1 staining^27^, a set of slides were incubated with the primary anti- anti-LIG1 rabbit monoclonal antibody (ab177946, ABCAM), incubated for 60 min at room temperature at 1:25 dilution. For LIG3^27^, TMA sections were overnight incubated at room temperature with 1:100 of anti-LIG3 rabbit polyclonal antibody (HPA006723, SIGMA). For PARP1^25^, a set of TMA sections were stained with mouse anti-human PARP1 antibody (1:600) for 60 min incubation in room temperature. For FEN1^26^, a set of slides were incubated with the primary anti-FEN1 mouse monoclonal antibody (NB100-150), at a dilution of 1:100, overnight at 4 °C. Sections were counterstained with haematoxylin. Cases with multiple cores were scored and the average was used as the final score. Negative (by omission of the primary antibody and IgG-matched serum) and positive controls (lymphnode/spleen) were included in each run.

### Evaluation of immune staining

Whole field inspection of the core was performed. The subcellular localisation of the marker was identified (nuclear, cytoplasm, cell membrane). Intensities of staining was assessed and grouped as follows: 0 = no staining, 1 = weak staining, 2 = moderate staining, 3 = strong staining. The percentage of tumour cell staining ranged from 0–100%. Histochemical score (H-score) (range 0–300) was calculated by multiplying the intensity of staining and the percentage of staining. A median H-score of ≤110 and ≤60 was used as the cut-off for high Mre11 nuclear and cytoplasmic expression respectively. Low/negative XRCC1 (XRCC1-) expression was defined by mean of H-score of ≤100^22^. Low/negative nuclear polβ (polβ-) expression was defined by median H-score of ≤180^21^. A median H score of <10 was utilised as the cut-off for low FEN1 nuclear expression^26^. For LIG1, low/negative nuclear expression was defined by H-score of < 60^21^. For LIG3, high nuclear was defined the H-score of > 0^27^. Median H score of <80 nuclear PARP1 staining was considered as low/negative^25^. A median H-score of ≤120 was used as the cut-off for low RAD50 nuclear expression^22^. A median H-score of ≤80 was used as the cut-off for low NBS1 nuclear expression^22^.

### Statistical analysis

Association with clinical and pathological parameters using categorised data was examined using Chi-squared test. All tests were 2-tailed. Survival rates were determined using Kaplan-Meier method and compared by the log-rank test. All analyses were conducted using Statistical Package for the Social Sciences (SPSS, version 22, Chicago, IL, USA) software for windows. *P* value of less than 0.05 was identified as statistically significant.

### *Mre11* transcript in ovarian cancers

Predictive and prognostic significance of *Mre11* mRNA expression (probe ID = 205395_s_at) was evaluated in a publicly available online gene expression dataset of 1259 ovarian cancer patients treated with platinum-based chemotherapy from 15 previously published studies^50^ and available at ‘http://kmplot.com/analysis/index.php?p=service&cancer=ovar’. The cut-off (Mre11 high or low) was based on median expression level.

Further Validation of Mre11 transcript as a predictive biomarker we utilized transcriptomic data from serous cystadenocarcinomas receiving platinum chemotherapy^51^. Box plot of pathological response and ROC curves were generated to evaluate the clinical utility of Mre11 as a predictive biomarker.

### Bioinformatics in ovarian cancer TCGA cohort

We next analysed RNA seq data from the ovarian serous cystadenocarcinoma TCGA cohort. *Mre11* was dichotomized into low (quartile 1) and high (quartile 4) based on normalized expression (FPKM) of *Mre11* obtained from the Xena browser^52^ and differentially expressed genes identified using DESeq2^53^. Genes were considered significantly differentially expression where fold changes ±2 and FDR < 0.05. Pathway analysis of significantly differentially expressed genes was conducted using WebGestalt^54^ to interrogate the KEGG database and chromosomal locations. Copy number variants were assessed using the cBioPortal^55^ and expression data in counts format was accessed using the GDC portal^56^.

### Cell lines and tissue culture

A2780 (platinum sensitive) and A2780cis (platinum resistant) were purchased from Sigma Aldrich (Gillingham, UK). PE01 (BRCA2-deficient, platinum sesnitive), PE04 (BRCA2-proficient, platinum resistant) were purchased from American Type Culture Collection (ATCC, Manassas, USA). Cells cultured in RPMI (R8758, Merck, UK) supplemented with 10% FBS (F4135, Merck, UK), 1% Penicillin-Streptomycin (P4333, Merck, UK). Hela-control, XRCC1 hela SilenciX were purchased from Tebu-Bio (www.tebu-bio.com). Hela control was cultured in Dulbecco’s Modified Eagle Medium (D6429, Sigma, UK) supplemented with 10% FBS and 1% penicillin streptomycin, while XRCC1 hela SilenciX were grown in Dulbecco’s Modified Eagle’s Medium-high glucose (D6429) supplemented with 10% FBS, 1% penicillin/streptomycin, and 125 μg/mL hygromycin B (H0654, Sigma,UK). All cell lines were maintained in a humidified incubator at 37 °C in a 5% CO2 atmosphere.

The breast cancer cell lines, MDA-MB-231, MCF7, MDA-MB-436 and MDA-MB-175 VII and SKBR3 were purchased from American Type Culture Collection (ATCC, Manassas, USA). MDA-MB-231 and MCF7 were grown in RPMI (R8758, Merck, UK). MDA-MB-436, was cultured in DMEM/F12 (ThermoFisher Scientific,11330032, UK) while MDA-MB-175 was cultured in Dulbecco’s Modified Eagle medium (DMEM) (Sigma-Aldrich, RNBH6547, UK).SKBR3 was cultured in McCoy’s medium (Sigma Life Science, SLCB4463, USA). All the mediums were supplemented with 10% foetal bovine serum (F4135, Merck, UK) and 1% Penicillin-Streptomycin (P4333, Merck, UK)

### Western Blot Analysis

Cells were harvested and lysed in RIPA buffer (R0278, Sigma.UK) with the addition of protease cocktail inhibitor (P8348, Sigma, UK), phosphatase inhibitor cocktail 2 (P5726, Sigma, UK) and phosphatase inhibitor cocktail 3 (P0044, Sigma) and stored at −20 °C. Proteins were quantified using BCA Protein Assay kit (23225, Thermofisher, UK). Samples were run on SDS-bolt gel (4–12%) bis-tris. Membranes were incubated with primary antibodies as follows: Mre11 (1:500, ab214), ß-actin (1:1000, ab8226), YY1 (1:1000, ab109228), GADPH (1:1000, ab9485), XRCC1 (1:1000, ab1838), pChk1 (1:1000, ab58567), Rad50 (1:1000, ab89), NBS1 (1:1000, Sigma, APREST78218), XRCC1 (1:500, ab1838), FEN-1 (1:1000, NB100-150), PARP (1:1000, 46D11 Cell Signaling), LIG1 (1:1000, ab177946), LIG3 (1:1000, HPA006723), RPA1 (1:1000, ab79398), RPA2 (1:1000, AB2175) and RPA3 (1:1000, ab97436). Membranes then were washed and incubated with Infrared dye-labelled secondary antibodies (LiCor) [IRDye 800CW Donkey Anti-Rabbit IgG (926-32213) and IRDye 680CW Donkey Anti-Mouse IgG (926-68072)] at dilution of 1:10,000 for 1 h. Membranes were scanned with a LiCor Odyssey machine (700 and 800 nm) to determine protein levels. Blots showed derived from the same experiments.

### Co-immunoprecipitation

Whole cell lysates of A2780, A2780cis, PEO1 and PEO4 were extracted. Cells were resuspended in RIPA buffer containing protease inhibitors on ice for 1 h. Lysates were incubated with the indicated antibodies overnight and then conjugated to protein A /G magnetic beads for 2 h at room temperature. After IP the beads were washed 4 times thoroughly with Phosphate buffer saline containing 0.01% Tween 20 and protease inhibitors. Immunoprecipitated proteins were eluted using 4x SDS loading buffer and then boiled at 100 ° Cfor 8 mins. Denaturated proteins were separated on 4–12% SDS PAGE.

### Nuclear and cytoplasmic extract

Cells were collected by trypsinization, washed with PBS and centrifuged at 1000 × *g* for 5 min. Nuclear and cytoplasmic lysates were extracted using the NE-PER Nuclear and Cytoplasmic Extraction Reagents (78833, Thermofisher, UK). Extracts were quantified using BCA protein quantification kit and protein levels were checked by western blot.

### Targeted next generation sequencing and bioinformatics

PicoPure^™^ DNA Extraction Kit (Thermofisher,UK) was used to extract genomic DNA from cell lines. Targeted next generation sequencing was investigated for genomic variants in A2780 and A2780cis cells. The SureSelect All Exon V5 kit (Agilent Technologies) was used to enrich for protein coding regions. The sequencing was performed using an Illumina NextSeq500 sequencer with paired end reads (150 bp) and a minimum of 88million reads generated per sample. Raw reads were then fastq formatted. Contaminating adapter sequences and low-quality sequences were later processed using Skewer^57^. Quality processed reads were then aligned to the HG19 reference genome using BWA^58^. Any duplicate alignments were identified and processed using PicardTools. Realignment was completed using the Abra assembly based realigner^59^ to allow insertion/deletion variants detection. Variant calling and filtering were performed with Samtools/Bcftools (v1.3.1)^60^. Using Vcftools^61^, variants, in variant call format (VCF), that associated with Platinum resistance were identified. The functional significance of annotated variants were assessed using the Ensembl Variant Effect Predictor tool^62^. Library preparation and sequencing was conducted by Source Biosciences (Nottingham, UK).

### Transient knockdowns of MRE11

Mre11 (ID S8960, sense sequence: CCCGAAAUGUCACUACUAATT and antisense sequence: UUAGUAGUGACAUUUCGGGAA) and the validation construct (Mre11 -S8959, sense sequence: GAUAGACAUUAGUCCGGUUTT and antisense sequence: AACCGGACUAAUGUCUAUCTT) siRNAs oligonucleotides were obtained from Invitrogen. Lipofectamine 3000 reagent (L3000015, Invitrogen, UK) was used according to the manufacturer’s protocol. Cells were platted overnight at 50–60% confluency in T25 flasks. Cells were then transfected with 20 nM of siRNA oligonuclotide or scrambled SiRNA oligonucleotide control (4390843, Thermofiher) in Opti-MEM media (31985-062, Gibco). Western blot was used to confirm transfection efficiency.

### CRISPR knock-out of XRCC1

Oligonucleotides carrying gRNA silencing XRCC1cloned in a Plv-U6g-EPCG plasmid (Sigma, UK) were transfected into A2780 cells. Cells were platted overnight at 50–60% confluency in 6 well plates. Cells were later transfected with 2–3 μg of DNA using Lipofectamine 3000 (Invitrogen, UK) in an Opti-MEM medium. Puromycin selection was started after 48 h for isolation of stable clones. Selection of A2780 cells was completed in 5 μg/ml puromycin for two weeks. Western blot was used to confirm the efficiency of XRCC1_KO. Multiple clones were selected and used in the current study.

### Clonogenic assays

In the clonogenic assay, 32 cells/cm^2^ were seeded in 6-well plates and left at 37 °C in a 5% CO_2_ atmosphere. Cisplatin (Kindly provided by Nottingham University Hospital) or Mirin (M9948, Sigma, UK) were added at the indicated concentrations and the plates were left at 37 °C in a 5% CO2 atmosphere for 14 days. Later the plates were washed with PBS, fixed and stained and colonies were counted.

### Generation of 3D spheroids

4 × 10^4^ cells per well were plated in ultra-low attachment 6-well plates in Promocell serum free tumour spheres medium (C-28070). Cells were then topped off with fresh medium every three days until spheroids structures were formed. Spheroids were treated with cisplatin or Mirin inhibitor for 48 h. To quantify cell viability, LIVE/DEAD Viability/Cytotoxicity Kit (L3224, Thermo Fisher Scientific) was used. Briefly, the spheroids were collected by trypsinization, washed with PBS and centrifuge at 1000 × *g* for 5 min. The light-protected cellular pellet in PBS was loaded with 0.1 μM of Calcein-AM and 1 μM of Ethidium homodimer-1 for 20 min at room temperature. The samples were then analysed on a Beckman-Coulter FC500 flow cytometer using a 495 nm laser for excitation and a 515 nm laser for emission data for Calcein AM and a 495 nm laser for excitation and emission at 635 nm for Ethidium Homodimer-1. In addition, Image J software was used to calculate spheroid diameter. Mean of three diagonal diameters was taken as diameter for each spheroid. At least 10 spheroids were measured.

### Functional studies

Functional studies were conducted for the accumulation of DNA double strand break, cell cycle progression and apoptosis assay using protocols described previously^63^. Briefly, 1 × 10^5^ cells per well were seeded overnight in 6- well plates at 37 °C in a 5% CO2 atmosphere. After 24 h, 5 µM of Cisplatin or 18 µM or 25 µM of Mirin were added to cells and incubated for 24 h and 48 h respectively. Cells were later collected by trypsinization, washed with ice cold PBS and fixed in 70% ethanol for 1 h at −20 °C. Fixative solution was later removed by centrifugation. For DNA double strand break analysis, cells were stained with 2 mg/ml of phospho-Histone (γH2AX) Ser139 (16202 A, Millipore, UK)^63^. For cell cycle analysis, cells were treated with 20 mg/ml RNase A (12091021, Invitrogen) and then 10 mg/ml Propidium Iodide (P4170, Sigma Aldrich) was added to determine the cell cycle distribution^63^. The samples were then analysed on a Beckman-Coulter FC500 flow cytometer using a 488 nm laser for excitation. The emission data for PI was collected using a 620 nm bandpass filter (FL3). The emission data for FITC-anti-phospho-Histone H2A.X was collected using a 525 nm bandpass filter (FL1). Apoptosis assay was completed using the Annexin V detection kit (556547, BD Biosciences). Briefly, cells were trypsinized, washed with PBS and the cellular pellet was re-suspended in Annexin Binding Buffer (1x). Then 2.5 ml of FITC Annexin V and 2.5 ml of Propidium Iodide were added to the cells. After incubation 300 ml of Annexin Binding Buffer (1x) was added to each tube. Samples were analysed on a Beckman-Coulter FC500 flow cytometer. Data were analysed by Weasel software. Graphical representation and statistical analysis were performed in GraphPad Prism 7 (GraphPad, La Jolla, USA).

### Docking of MRE11 inhibitor (Mirin) in crystal structure of Mre11

Structure of human Mre11 (PDB: 3t1i, PMID 20122942) was prepared for docking by removal of solvent (DTT, glycerol and water) and superposed to the TmMRE11:mirin complex (PDB: 4o4k, PMID 24316220). See results section for full details.

### Reporting summary

Further information on research design is available in the Nature Research Reporting Summary linked to this article.

## Supplementary information

REPORTING SUMMARY

Supplementary information

Supplementary Data 1

Supplementary Data 2

## Data Availability

Data supporting the study can be found in the supplementary information file, and the corresponding author can make any materials available upon request. Aggregate data from the referenced datasets are available from the corresponding author on reasonable request. Primary datasets generated during the study are available in supplementary tables 7 and 8. Referenced datasets analyzed in the study are described in methods and accession codes are as follows; GSE14764, GSE15622, GSE19829, GSE3149, GSE9891, GSE18520, GSE26712, and TCGA (The Cancer Genome Atlas). The cancer genome atlas (TCGA) ovarian cancer RNAseq gene expression data was accessed from the NCI Genomics Data Commons at the following link: https://portal.gdc.cancer.gov/repository. The full NGS data has been uploaded and is available at NCBI-GEO (https://www.ncbi.nlm.nih.gov/geo/) with the following accession numbers GSE198648 (PEO1, PEO1R cell lines) and GSE160540 (PEO1, PEO1R xenografts).
