# Supplementary material for: Targeting Mre11 overcomes platinum resistance and induces synthetic lethality in XRCC1 deficient epithelial ovarian cancers
Source: NPJ Precis Oncol. 2022 Jul 19;6:51. doi: 10.1038/s41698-022-00298-0 (PMC9296550; doi:10.1038/s41698-022-00298-0)
Supplement: Supplementary file 1 — REPORTING SUMMARY [file 41698_2022_298_MOESM1_ESM.pdf]

nature research

Corresponding author(s): Prof Srinivasan Madhusudan (Manuscript ID-NPJPRECISIONONCOLOGY-01711)

Reporting Summary

Nature Research wishes to improve the reproducibility of the work that we publish. This form provides structure for consistency and transparency in reporting. For further information on Nature Research policies, see Authors & Referees and the Editorial Policy Checklist.

Statistical parameters

When statistical analyses are reported, confirm that the following items are present in the relevant location (e.g. figure legend, table legend, main text, or Methods section).

n/a	Confirmed	
☐	☒	The exact sample size (*n*) for each experimental group/condition, given as a discrete number and unit of measurement
☒	☐	An indication of whether measurements were taken from distinct samples or whether the same sample was measured repeatedly
☐	☒	The statistical test(s) used AND whether they are one- or two-sided<br>*Only common tests should be described solely by name; describe more complex techniques in the Methods section.*
☐	☒	A description of all covariates tested
☒	☐	A description of any assumptions or corrections, such as tests of normality and adjustment for multiple comparisons
☐	☒	A full description of the statistics including central tendency (e.g. means) or other basic estimates (e.g. regression coefficient) AND variation (e.g. standard deviation) or associated estimates of uncertainty (e.g. confidence intervals)
☒	☐	For null hypothesis testing, the test statistic (e.g. *F*, *t*, *r*) with confidence intervals, effect sizes, degrees of freedom and *P* value noted<br>*Give P values as exact values whenever suitable.*
☒	☐	For Bayesian analysis, information on the choice of priors and Markov chain Monte Carlo settings
☒	☐	For hierarchical and complex designs, identification of the appropriate level for tests and full reporting of outcomes
☒	☐	Estimates of effect sizes (e.g. Cohen's *d*, Pearson's *r*), indicating how they were calculated
☐	☒	Clearly defined error bars<br>*State explicitly what error bars represent (e.g. SD, SE, CI)*

Our web collection on statistics for biologists may be useful.

Software and code

Policy information about availability of computer code

Data collection	Transcriptomic analyses was completed with an online platforms<br>1. http://kmplot.com/analysis/index.php?p=service&cancer=ovar<br><br>Detailed bio-informatics was completed using the TCGA ovarian cancer dataset available at:<br>https://www.cancer.gov/about-nci/organization/ccg/research/structural-genomics/tcga
Data analysis	Transcriptomic analyses was completed with an online platforms<br>1. http://kmplot.com/analysis/index.php?p=service&cancer=ovar<br><br>Detailed bio-informatics was completed using the TCGA ovarian cancer dataset available at:<br>https://www.cancer.gov/about-nci/organization/ccg/research/structural-genomics/tcga

For manuscripts utilizing custom algorithms or software that are central to the research but not yet described in published literature, software must be made available to editors/reviewers upon request. We strongly encourage code deposition in a community repository (e.g. GitHub). See the Nature Research guidelines for submitting code & software for further information.

Data

Policy information about availability of data

All manuscripts must include a data availability statement. This statement should provide the following information, where applicable:

- Accession codes, unique identifiers, or web links for publicly available datasets
- A list of figures that have associated raw data
- A description of any restrictions on data availability

Data supporting the study can be found in the supplementary information file, and the corresponding author can make any materials available upon request. Aggregate data from the referenced datasets are available from the corresponding author on reasonable request. Primary datasets generated during the study are available in supplementary tables 7 and 8. Referenced datasets analyzed in the study are described in methods and accession codes are as follows; GSE14764, GSE15622, GSE19829, GSE3149, GSE9891, GSE18520, GSE26712, and TCGA.

Field-specific reporting

Please select the best fit for your research. If you are not sure, read the appropriate sections before making your selection.

☒ Life sciences ☐ Behavioural & social sciences ☐ Ecological, evolutionary & environmental sciences

For a reference copy of the document with all sections, see nature.com/authors/policies/ReportingSummary-flat.pdf

Life sciences study design

All studies must disclose on these points even when the disclosure is negative.

Sample size	Investigation of the expression of Mre11 was carried out on tissue microarrays of 331 consecutive ovarian epithelial cancer
Data exclusions	None
Replication	All western blots were performed in triplicates
Randomization	Not applicable
Blinding	Not applicable

Reporting for specific materials, systems and methods

Materials & experimental systems

n/a	Involved in the study
☒	☐ Unique biological materials
☐	☒ Antibodies
☐	☒ Eukaryotic cell lines
☒	☐ Palaeontology
☐	☐ Animals and other organisms
☐	☒ Human research participants

Methods

n/a	Involved in the study
☒	☐ ChIP-seq
☒	☐ Flow cytometry
☒	☐ MRI-based neuroimaging

Antibodies

Antibodies used	TMAs were constructed and immunohistochemically profiled for MRE11. A set of slides were incubated with the primary anti-Mre11 mouse monoclonal antibody (clone ab214, Abcam), at a dilution of 1:800, for 1 hour at room temperature.
Validation	We validated MRE11 in a panel of ovarian cancer cell lines . In addition, transient knock down of MRE11 using siRNAs have been performed to confirm the validity of the antibody used in the current IHC study. Negative controls for IHC included omission of the primary antibody and IgG-matched serum. Positive control included normal lymphoid (Lymph Node/spleen) tissue within the TMA.

Eukaryotic cell lines

Policy information about cell lines

Cell line source(s)	A2780 and A2780cis cell lines were purchased from Sigma Aldrich (Gillingham, UK). PE01, PE04, MDA-MB-231, MCF7, MDA-MB-436 and MDA-MB-175 VII and SKBR3 were purchased from ATCC. Hela-control, XRCC1 hela SilenciX were purchased from Tebu-Bio (www.tebu-bio.com).
Authentication	STR profiling
Mycoplasma contamination	All cell lines were routinely tested for mycoplasma on a 3 monthly basis
Commonly misidentified lines (See ICLAC register)	None

Animals and other organisms

Policy information about studies involving animals; ARRIVE guidelines recommended for reporting animal research

Laboratory animals	na
Wild animals	na
Field-collected samples	*For laboratory work with field-collected samples, describe all relevant parameters such as housing, maintenance, temperature, photoperiod and end-of-experiment protocol OR state that the study did not involve samples collected from the field.*

Human research participants

Policy information about studies involving human research participants

Population characteristics	Investigation of the expression of Mre11 was carried out on tissue microarrays of 331 consecutive ovarian epithelial cancer cases treated at Nottingham University Hospitals (NUH) between 1997 and 2010. This study was carried out in accordance with the declaration of The Helsinki and ethical approval which was obtained from the Nottingham Research Ethics Committee (REC Approval Number 06/Q240/153).
Recruitment	None
