# Supplementary material for: Targeting Mre11 overcomes platinum resistance and induces synthetic lethality in XRCC1 deficient epithelial ovarian cancers
Source: NPJ Precis Oncol. 2022 Jul 19;6:51. doi: 10.1038/s41698-022-00298-0 (PMC9296550; doi:10.1038/s41698-022-00298-0)

Supplementary Table 1: Patient demographics and pathological features in ovarian cancer cohort.

	**Number**	**Percentages**
Pathology		
Serous cystadenocarcinoma	178	53.9%
Endometrioid	44	13.2%
Clear cell carcinoma	24	7.2%
Mucinous cystadenocarcinoma	46	13.8%
Others	15	4.5%
Mixed	17	5.1%
missing	7	2.3%
Grade		
1	46	13.8%
2	60	18%
3	171	51.5%
missing	54	16.7%
Residual tumors		
None/Microscopic	205	61.7%
<1cm	34	10.2%
>1-2 cm	15	4.5%
>2cm	40	12%
Missing	37	11.6%
FIGO Stage		
I	123	37%
II	49	14.7%
III	128	38.5%
IV	11	3.3%
Missing	20	6.5%
Chemotherapy		
Carboplatin monotherapy	100	30%
Carboplatin + Paclitaxel	110	33%
Other regimens *	26	8%
No chemo	23	7 %
unknown	72	22%
Platinum sensitivity		
Sensitive **	250	75.6%
Resistant	26	7.8%
Unknown	55	16.6%
Relapse status		
Progression-free ***	169	50.9%
Progressed/relapsed	118	35.6%
Unknown	44	13.3%

Other Regimens included: 3= CAP (Cyclophosphamide, Adriamycin and Cisplatin), 4 = ICON5 Trial, 5 = SOCTROC Trial, 6 = Carboplatin and Endoxan and 7 = Chlorambucil. For

the unknown category, the majority received platinum-based chemo but because the exact regimen was not mentioned clearly in the database, they were considered as unknown. However, for these cases the platinum sensitivity status was clearly mentioned / recorded.
** Platinum resistance was defined as patients who had progression during first-line platinum chemotherapy or relapse within 6 months after completion of platinum treatment.
*** Progression-free survival was calculated from the date of the initial surgery to disease progression or from the date of the initial surgery to the last date known to be progression-free at the end of follow-up. All the patients' dates of disease recurrence were recorded in the main sheet where we used to perform the analysis.

Supplementary Table 2: MRE11 nuclear expression and ovarian cancers

	**MRE11(nuc) -**	**MRE11(nuc) +**	**P- value**
Pathological Type			***0.000073***
Serous adenocarcinoma	49 (36%)	87 (64%)	
Mucinous adenocarcinoma	23 (82.1%)	5 (17.9 %)	
Endometrioid carcinoma	15 (50%)	15 (50%)	
Clear cell carcinoma	10 (76.9%)	3 (23.1%)	
Mixed	4 (33.3 %)	8 (66.7 %)	
FIGO Stage			***0.020***
I	50 (58.8%)	35 (41.2%)	
II	13 (37.1%)	22 (62.9%)	
III	37 (39.4%)	57 (60.6%)	
IV	2 (25%)	6 (75%)	
Tumour Grade			0.118
Grade 1	17 (63%)	10 (37%)	
Grade 2	17 (39.5%)	26 (60.5)	
Grade 3	54 (42.9%)	72 (57.1)	
Surgical Optimal Debulking			***0.002***
Optimally Debulked	87 (51.8%)	81 (48.2%)	
Not Optimally Debulked	10 (25.6%)	29 (74.4%)	
Residual tumour			0.16
None/Microscopic	71 (22.7%)	64 (47.4%)	
<1cm	12 (41.4%)	17 (58.6%)	
1-2 cm	5 (41.7%)	7 (58.3%)	

>2cm	9 (31%)	20 (69%)	
Measurable Disease Before Chemotherapy			0.06
Non	69 (50%)	69 (50%)	
Measurable	24 (37.5%)	40 (62.5%)	

Supplementary Table 3: MRE11 cytoplasmic expression and ovarian cancers

	**MRE11 (cyto) -**	**MRE11(cyto) +**	**P- value**
Pathological Type			***0.000046***
Serous adenocarcinoma	25 (38.2%)	84 (61.8%)	
Mucinous adenocarcinoma	22 (78.6%)	6 (22.4%)	
Endometrioid carcinoma	11 (36.7%)	19 (63.3%)	
Clear cell carcinoma	12 (92.3%)	1 (7.7%)	
Mixed	6 (50%)	6 (50%)	
FIGO Stage			0.113
I	48 (56.5%)	37 (43.5%)	
II	17 (48.6%)	18 (51.4%)	
III	36 (38.3%)	58 (61.7)	
IV	4 (50%)	4 (50%)	
Tumour Grade			***0.007***
Grade 1	19 (70.4)	8 (29.6%)	
Grade 2	23 (53.5%)	20 (46.5%)	
Grade 3	49 (38.9%)	77 (61.1%)	
Surgical Optimal Debulking			0.072
Optimally Debulked	89 (53%)	79 (47%)	
Not Optimally Debulked	15 (38.5%)	24 (61.5%)	
Residual tumour			0.137
None/Microscopic	76 (56.3%)	59 (43.7%)	
<1cm	10 (34.5%)	19 (65.5%)	
1-2 cm	5 (34.5%)	7 (58.3%)	
>2cm	13 (44.8%)	16 (55.2)	

Measurable Disease Before Chemotherapy			0.19
Non	73 (52.9%)	65 (47.1%)	
Measurable	29 (45.3%)	35 (54.7%)	

Supplementary Table 4: Multivariate analysis for progression free survival (PFS)

**Variables**	**Beta**	**p-value**	**Risk ratio**	**Risk ratio 95% lower**	**Risk ratio 95% upper**
Surgical Pathology stage	0.567	**0.0001**	1.763	1.330	2.336
Surgical Pathology grade	0.052	0.787	1.053	0.724	1.531
Age	-0.310	0.181	0.733	0.466	1.155
Mre11 (nuc) expression	0.004	**0.019**	1.004	1.0	1.007

Supplementary Table 5: Multivariate analysis for overall survival (OS)

**Variables**	**Beta**	**p-value**	**Risk ratio**	**Risk ratio 95% lower**	**Risk ratio 95% upper**
Surgical Pathology stage	0.592	**0.0001**	1.808	1.436	2.277
Surgical Pathology grade	-0.154	0.287	0.857	0.645	1.139
Age	0.349	0.081	1.418	0.958	2.098
Mre11 (nuc) expression	0.003	**0.029**	1.003	1.0	1.006

Supplementary Table 6: Mre11 and other DNA repair markers.

MRE11(nuclear) Expression			
**Variable**	**Low**	**High**	***P*- value**
	***N* (%)**	***N* (%)**	
**RAD50**			
Low	73 (67)	36 (33)	**<0.001**
High	25 (26)	71 (74)	
**NBS1**			
Low	95 (62)	58 (38)	**<0.001**
High	5 (9)	50 (91)	
**XRCC1**			
Low	49 (91)	5 (9)	**<0.001**
High	60 (34)	114 (66)	
**LIG3**			
Low	71(74)	25(26)	**<0.001**
High	36(28)	92 (71.9)	
**LIG1**			
Low	66 (67)	33 (33)	**<0.001**
High	41 (32)	86 (68)	
**FEN1**			
Low	66 (62)	40 (38)	**<0.001**
High	37 (33)	76 (67)	
**Pol β**			
Low	78 (67)	39 (33)	**<0.001**
High	24 (24)	75 (76)	
**PARP1**			
Low	46 (71)	19 (29)	**<0.001**
High	45 (35)	82 (65)	

Supplementary Table 7. Pathway analysis of TCGA Ovarian serous cystadenocarcinomas

Gene Set	Description	Size	Expect	Ratio	P Value	FDR
hsa05322	Systemic lupus erythematosus	133	1.8109	5.5221	1.17E-05	0.003388
hsa00590	Arachidonic acid metabolism	63	0.85779	8.1605	2.08E-05	0.003388
hsa00480	Glutathione metabolism	56	0.76248	7.869	0.000102	0.011137
hsa05034	Alcoholism	180	2.4508	4.0802	0.000156	0.012702
hsa00983	Drug metabolism	79	1.0756	5.5781	0.000682	0.044476

Supplementary Table 8: MRE11 interactors in A2780cis cells

Gene	Transcript	Variant Type	Variant	Amino Acid Position	Sample
ATM	ENST00000278616.4	Synonymous	caG/caA	Q2220	A2780cis
C15orf26	ENST00000286732.4	Synonymous	aaG/aaA	K35	A2780cis
NBN	ENST00000265433.3	Missense	Gga/Aga	G214R	A2780cis
NBN	ENST00000265433.3	Missense	Aat/Tat	N127Y	A2780cis
RBBP8	ENST00000327155.5	Missense	aTt/aAt	I796N	A2780cis
RBBP8	ENST00000583057.1	Missense	Ttg/Atg	L221M	A2780cis
RAD50	ENST00000265335.6	Missense	gCt/gAt	A1175D	A2780cis

Supplementary Figures

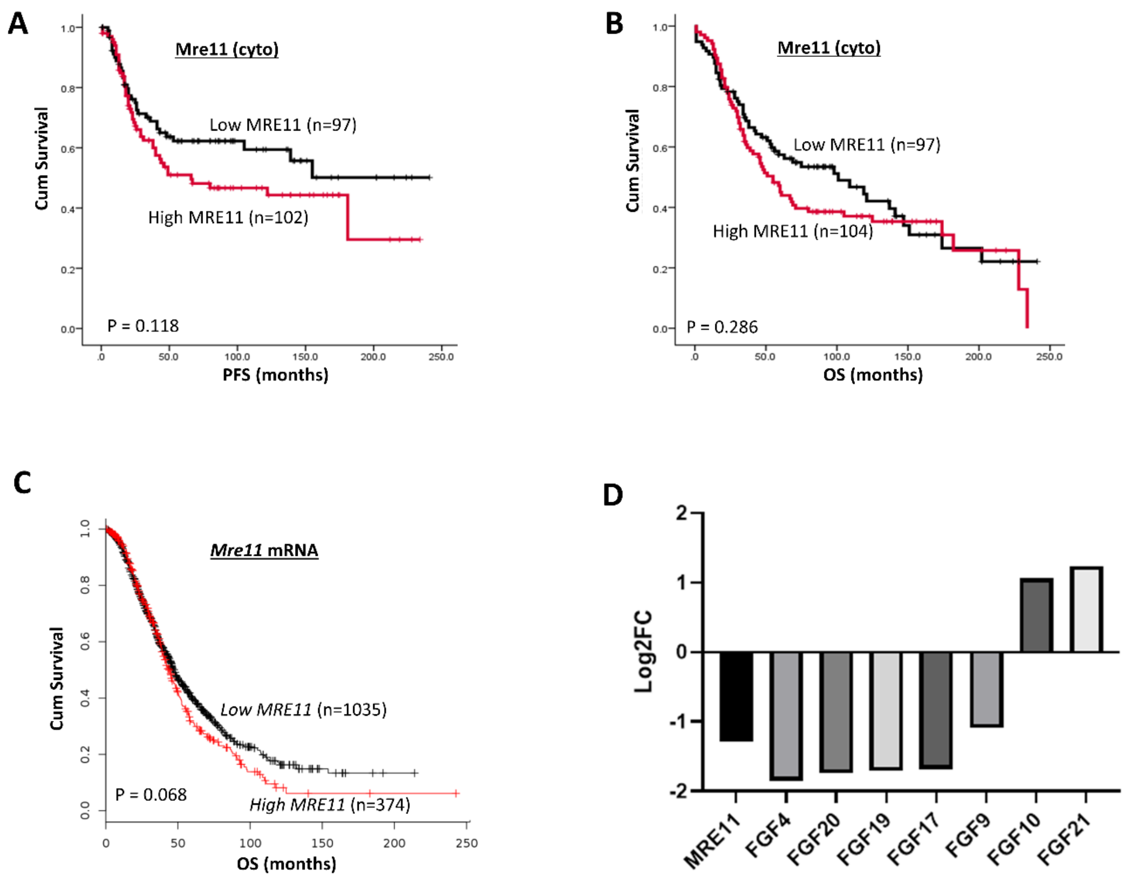

Supplementary Figure 1: **MRE11 expression and ovarian cancer.** (**A**) Kaplan-Meier curve for Mre11 cytoplasmic expression and progression free survival (PFS) in ovarian cancers. (**B**) Kaplan-Meier curve for Mre11 cytoplasmic expression and overall survival (OS) in ovarian cancers. (C) Kaplan Meier curve for *Mre11* mRNA expression and overall survival (OS) in ovarian cancers. (D) TCGA RNAseq analysis of selected markers in 325 tumours that had low *Mre11* expression.

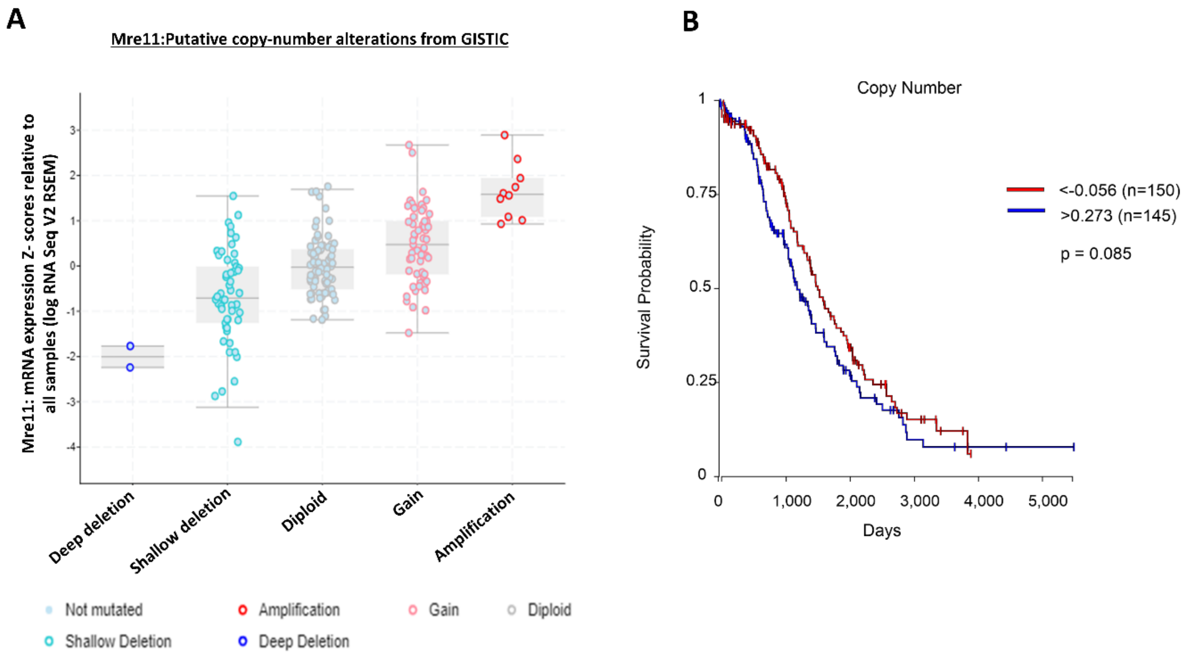

Supplementary Figure 2: MRE11 gene copy number changes and ovarian cancers. (A) Mre11 gene copy number changes and mRNA levels. Box Plot- lower whisker [Minimum (Q0 or 0th percentile)] is the lowest data point in the data set excluding any outliers. Upper whisker [Maximum (Q4 or 100th percentile)] is the highest data point in the data set excluding any outliers. Central line [Median (Q2 or 50th percentile)]is the middle value in the data set. Bounds of the box - First quartile (Q1 or 25th percentile) is the median of the lower half of the dataset. Third quartile (Q3 or 75th percentile) is the median of the upper half of the dataset. The box is drawn from Q1 to Q3 with a horizontal line drawn in the middle to denote the median. **(B)** Kaplan-Meier curve for Mre11 gene copy number changes and survival.

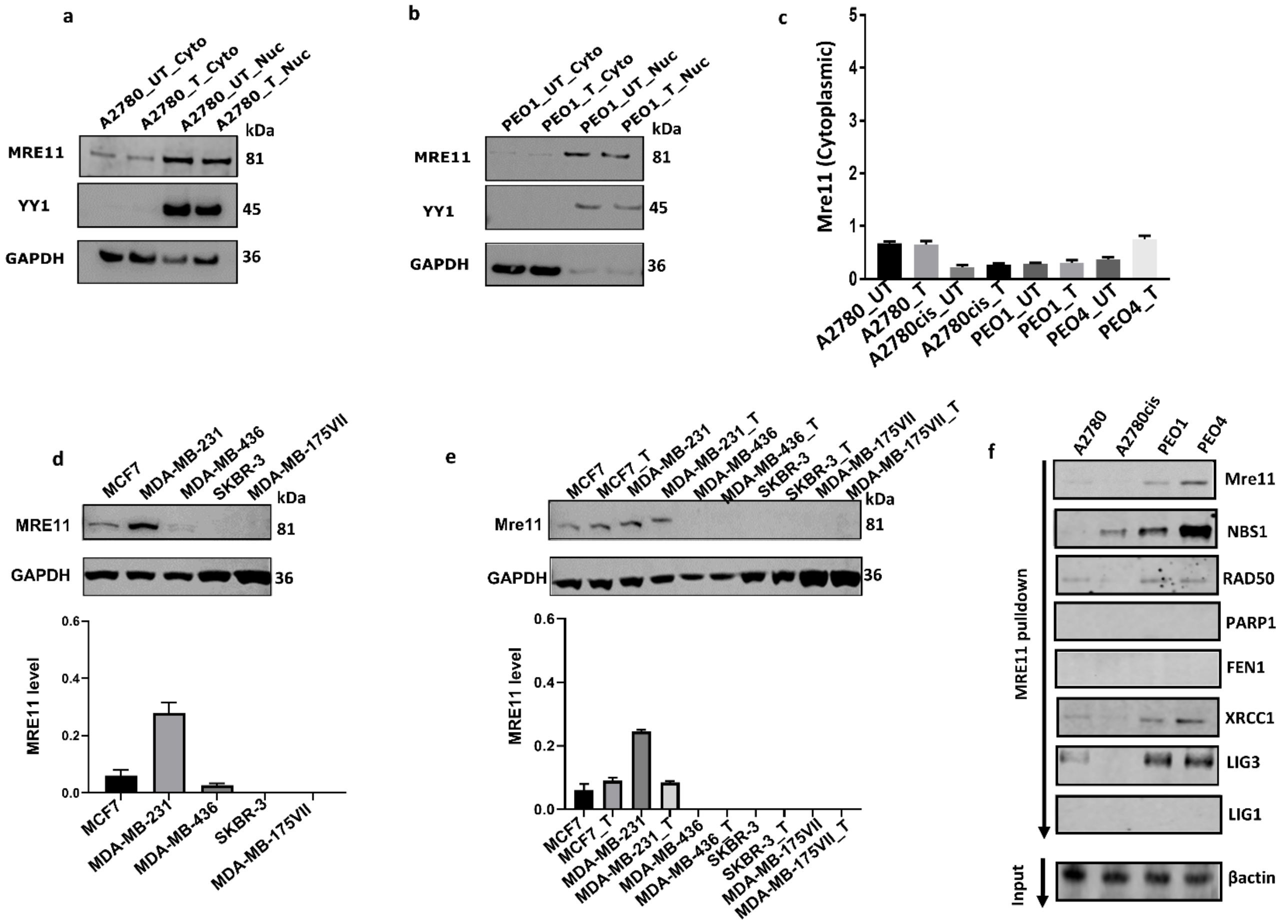

Supplementary Figure 3: MRE11 expression in nuclear and cytoplasmic extracts of ovarian cancer cell lines. (A) MRE11 levels in nuclear and cytoplasmic extracts of A2780 cells treated with cisplatin for 24 h.(**B**) MRE11 levels in nuclear and cytoplasmic extracts of PEO1 cells treated with cisplatin for 24 h. (**C**) Quantification of MRE11 protein levels by western blot in cytoplasmic extracts of A2780, A2780cis, PEO1 and PEO4 treated with 5μM cisplatin for 24 h. **(D)** Mre11 level at baseline in a panel of breast cancer cell lines. **(E)** Mre11 level, pre and post cisplatin treatment in a panel of breast cancer cell lines. **(F)** Co-immunoprecipitation of Mre11 with other DNA repair markers. See methods for details. Figures are representative of three or more independent experiments. Error bars represent standard error of mean between experiments. All western blots were derived from the same experiment and were processed in parallel.

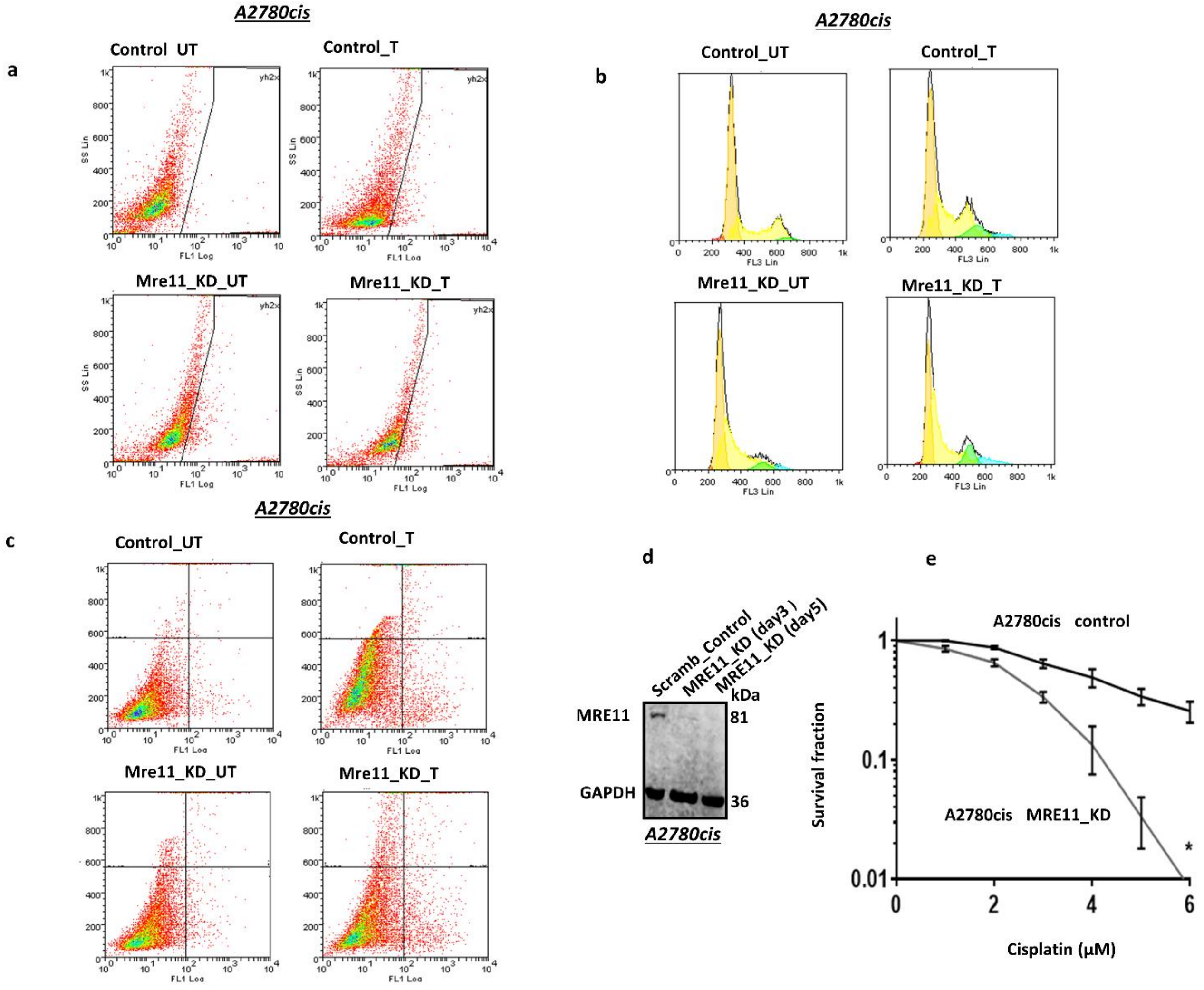

Supplementary Figure 4: Functional analysis in control and MRE11 knockout ovarian cancer cells. (**A)** Representative images for γH2AX analysis by weasel software. (**B**) Representative images for cell cycle analysis by weasel software (**C)** Representative images for AnnexinV analysis by weasel software. (**D**) Western blot for MRE11 knockdown in A2780cis cells. (**E)** Clonogenic survival assay for cisplatin sensitivity in A2780cis control and MRE11 knock down cells. Figures are representative of three or more independent experiments**.** Error bars represent standard error of mean between experiments. All western blots were derived from the same experiment and were processed in parallel.

Full uncropped gels

Supplementary Figure 3

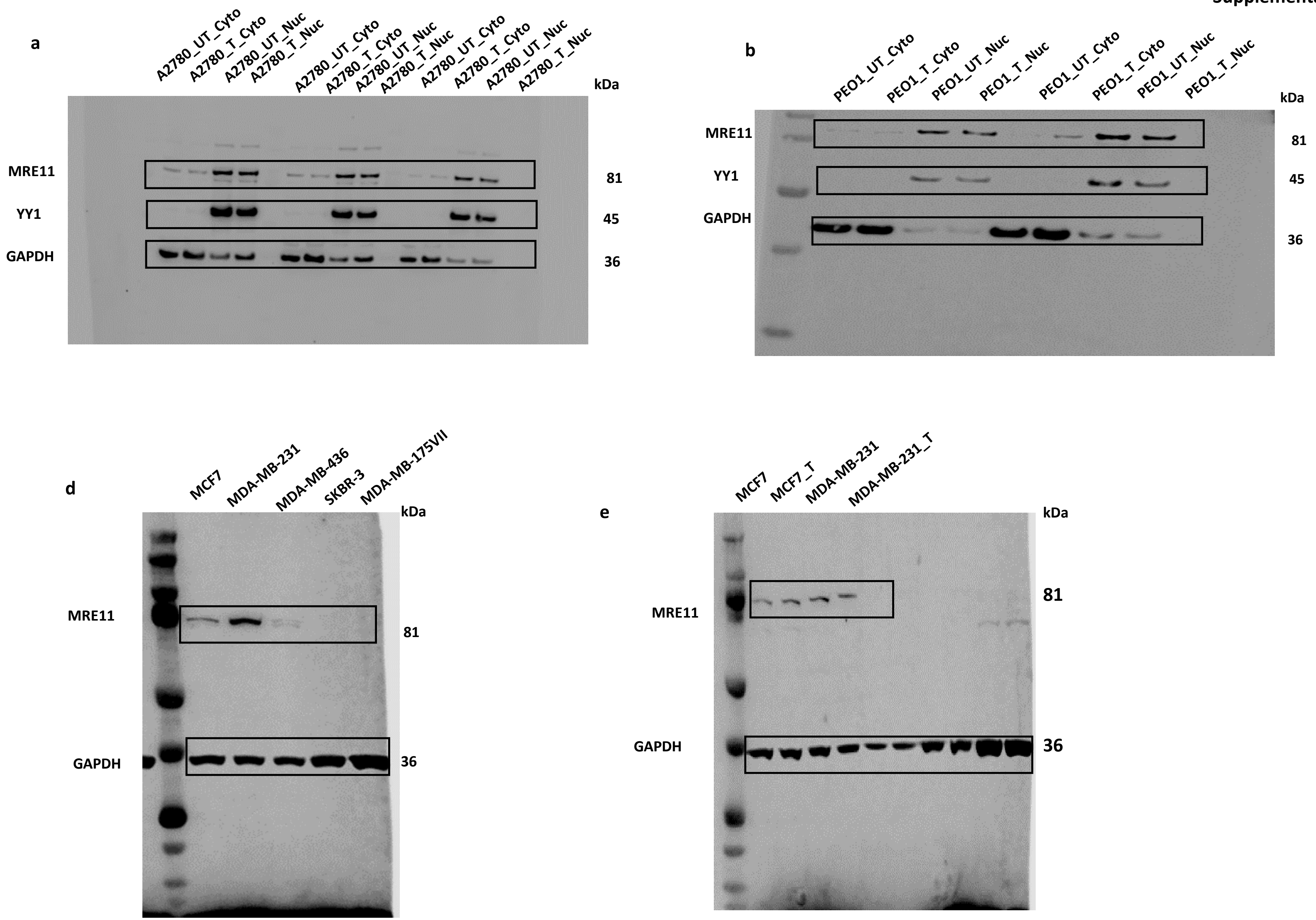

f

MRE11 BLOT

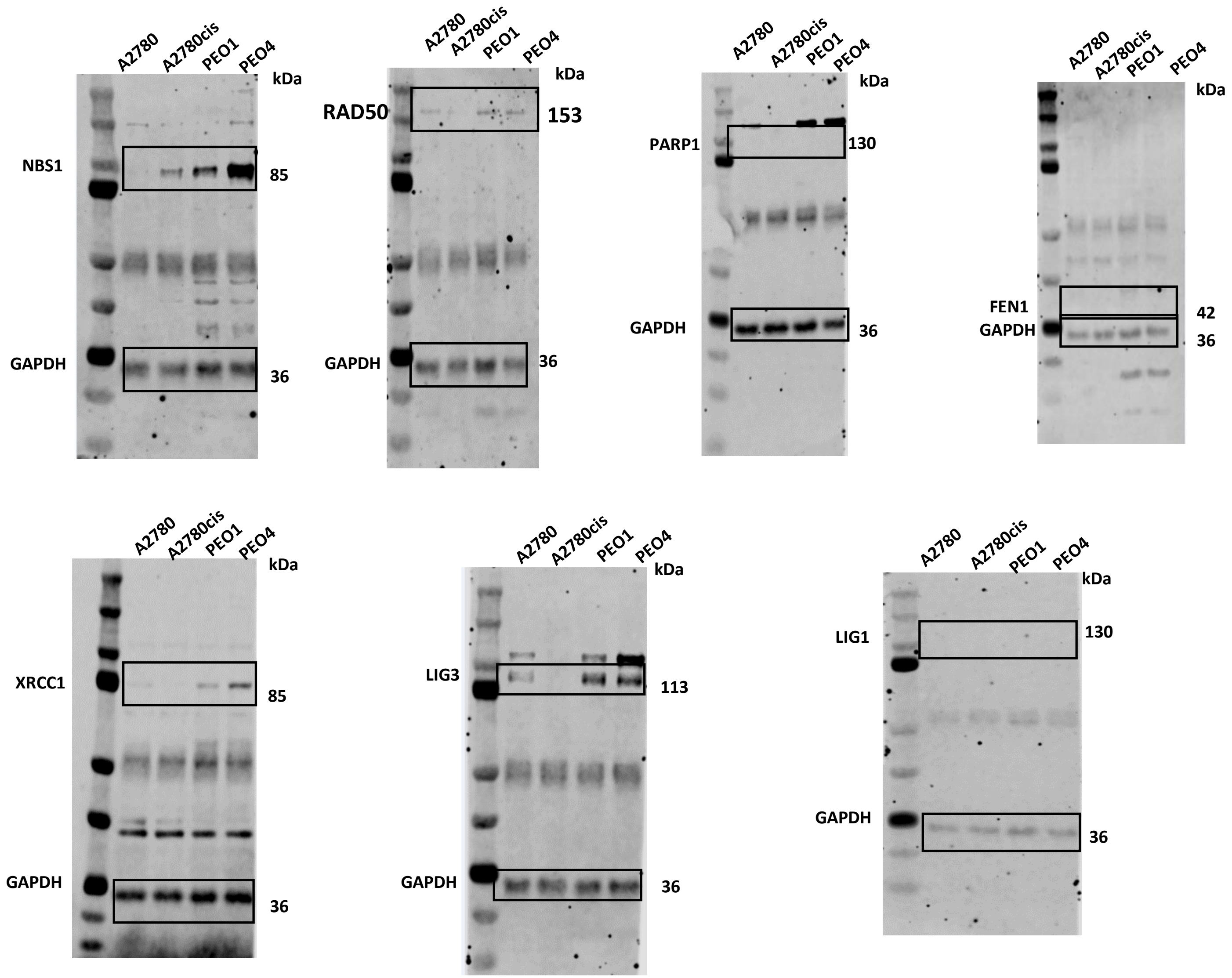
A2780
A2780cis
PEO1
PEO4
kDa
NBS1
85
GAPDH
36
RAD50
153
36
PARP1
130
36
FEN1
42
36
XRCC1
85
LIG3
113
LIG1
130

d

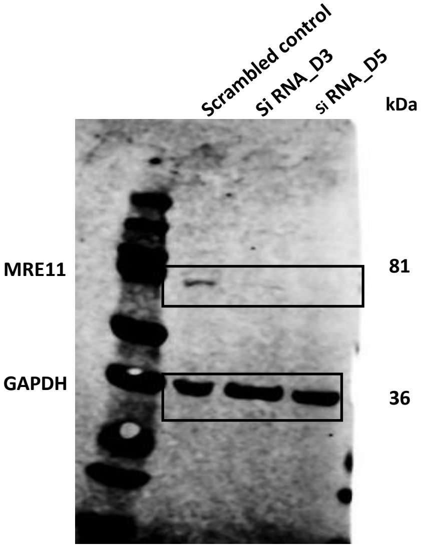

Supplement: Supplementary file 2 — Supplementary information [file 41698_2022_298_MOESM2_ESM.pdf]
